# The changing face of acute low back pain management by physiotherapists, osteopaths and chiropractors in the UK: a 20-year comparison from 2003 to 2023

**DOI:** 10.1186/s12891-025-09192-9

**Published:** 2025-10-02

**Authors:** David W. Evans, Nadine E. Foster, Alan C. Breen, Tamar Pincus, Martin Underwood, Steven Vogel

**Affiliations:** 1 School of Sport, Exercise and Rehabilitation Sciences, University of Birmingham, Edgbaston, Birmingham, B15 2TT UK; 2 STARS Education and Research Alliance, Surgical Treatment and Rehabilitation Service (STARS), The University of Queensland and Metro North Health, Brisbane, QLD 407206 Australia; 3 Health Sciences University, Bournemouth Campus, Parkwood Road, Bournemouth, BH5 2DF UK; 4 School of Psychology, University of Southampton, University Road, Southampton, SO17 1BJ UK; 5 Warwick Clinical Trials Unit, Warwick Medical School, The University of Warwick, Coventry, CV4 7AL UK; 6 Health Sciences University, London Campus, 275 Borough High Street, London, SE1 1JE UK

**Keywords:** Low back pain, Musculoskeletal, Clinical behavior, Physiotherapists, Chiropractors, Osteopaths

## Abstract

**Background:**

Low back pain (LBP) remains a leading source of disability and societal cost. In the UK, physiotherapists, osteopaths, and chiropractors are front-line providers of LBP care. Despite widespread dissemination of clinical guidelines, little is known about how their clinical practice has changed over time.

**Methods:**

We conducted national surveys of UK physiotherapists, osteopaths and chiropractors in 2003 and 2023, using an identical acute non-specific LBP vignette to assess reported use of investigations and interventions at both time points.

**Results:**

A total of 1,758 eligible clinicians participated in 2003 (834 physiotherapists; 592 osteopaths; and 332 chiropractors), and 1,388 in 2023 (511 physiotherapists; 621 osteopaths; and 255 chiropractors). At both time-points, there were significant inter-professional differences and numerous departures from guideline recommendations. Substantial temporal changes were observed. Physiotherapists shifted towards more restrictive recommendations for work and bed-rest, towards the use of massage, away from spinal mobilization, away from specific exercises, and away from general advice on back care. Chiropractors shifted towards more restrictive recommendations for bed-rest, towards spinal mobilization and acupuncture, and away from spinal manipulation and ultrasound. Osteopaths shifted towards less restrictive recommendations for activity, work, and bed-rest, towards acupuncture, and away from spinal manipulation.

**Conclusions:**

Between 2003 and 2023, UK physiotherapists, osteopaths, and chiropractors reported evolving management approaches to acute LBP. Substantial inter-professional differences and divergences from guideline recommendations were observed. Some inter-professional differences narrowed over time, suggesting partial convergence of practice.

**Supplementary Information:**

The online version contains supplementary material available at 10.1186/s12891-025-09192-9.

## Contributions to the literature

- Despite multiple iterations of clinical guidelines for low back pain, little is known about how practice has evolved among front-line musculoskeletal clinicians.
- This study offers a unique 20-year comparison of reported clinical practice among physiotherapists, osteopaths, and chiropractors in the UK, using identical surveys at both time points.
- Results show ongoing differences between professional groups and gaps between guidelines and practice, but also clear evidence that practice has evolved unevenly, with some signs of increasing agreement over time.
- This study provides new evidence on how guideline-based care is adopted – or not – across related healthcare professions in real-world settings.

## Introduction

Despite decades of research, multiple iterations of clinical guidelines [1–3], and widespread implementation efforts [4–8], low back pain (LBP) continues to be a leading source of disability globally that results in personal suffering for millions and enormous societal costs [9]. The high-profile 2018 *Lancet Series* on LBP [10] drew attention to an international evidence-practice gap, highlighting that interventions widely recommended as first-line treatments were underutilised [11, 12]. It also called for greater promotion of activity, including work participation, and recommended behaviour change amongst clinicians [12, 13]. Although healthcare regulators worldwide have largely adopted policies for LBP that endorse the provision of advice to stay active and the judicious use of medications, imaging and surgery, the use of these approaches in everyday clinical practice remains largely unknown [11–13].

In the UK, the majority of LBP care takes place in primary care, spanning both government-funded National Health Service (NHS) settings and private practice [14]. Central to this care are three professional groups: physiotherapists, osteopaths, and chiropractors. Although these groups share similar clinical aims for LBP patients, they have historically differed to varying degrees in their conceptual frameworks [15, 16], preferred assessment approaches [17–19], and treatments [14, 20–22]. Over the last two decades, multiple factors – such as prominent best-practice guidelines [23–26], heightened attention to patient safety [27–30], and evolving professional regulation [31–36] – have been designed to influence the behaviour of these clinicians regarding LBP. However, it remains unclear whether changes in clinical practice have occurred and, if so, whether they have occurred consistently across all three professional groups.

Direct comparisons of how UK physiotherapists, osteopaths, and chiropractors care for LBP are lacking, making it difficult to ascertain whether these groups are converging on or diverging from recommended approaches. Generating multiple comparable snapshots of clinical behaviour over a long period (i.e., decades) would offer an opportunity to observe whether – and how – the LBP care offered by these professional groups is evolving and whether inter-professional differences are reducing or increasing. Against this backdrop, the aims of this study were to: (1) report snapshots of clinical investigations and interventions selected for the same acute non-specific LBP scenario by UK physiotherapists, osteopaths, and chiropractors in 2003 and 2023 respectively; (2) identify similarities and differences in this reported behaviour between professional groups at each of these time points; and (3) estimate how this reported behaviour has changed within each professional group over this 20-year period.

## Methods

We recruited national samples of physiotherapists, osteopaths and chiropractors, working in the UK and managing patients with LBP, to take part in separate surveys in 2003 and 2023. Informed consent was provided by all participants. Ethical approval was provided in 2003 by the London Multicentre Research Ethics Committee (ref: MREC/03/2/045). In 2023, ethical approval was provided by the University of Birmingham Science, Technology, Engineering and Mathematics Research Ethics Committee (ref: ERN_0530-Jul2023). All procedures were conducted in accordance with the Helsinki Declaration of 1975, as revised in 1983.

### Recruitment and data collection (2003)

All 2003 data were collected using a printed postal survey, which formed the baseline data of a large randomised controlled trial evaluating the effect of printed educational material, containing recommendations from LBP clinical guidelines of the time, on the reported behaviour and beliefs of clinicians [37]. Full methodological details of this trial have been published previously [38]. Importantly, these baseline data were collected prior to any exposure to the educational material and can therefore be treated as a cross-sectional survey of clinicians’ reported behaviours and beliefs at the time.

To recruit nationally proportional samples of physiotherapists, osteopaths, and chiropractors in 2003, ~ 50% of eligible clinicians were randomly selected and invited from each professional register [38]. Invitations were sent directly to participants using registrant postal addresses provided upon request from: the Chartered Society of Physiotherapy (physiotherapists who had designated their specialty as ‘musculoskeletal’); the General Osteopathic Council (osteopaths); and the British Chiropractic Association (chiropractors). In 2003, clinicians with contact addresses outside of the UK or a Crown Dependency were excluded before the sampling frame was drawn. Likewise, all practitioners living or working in Scotland were excluded in 2003 due to the likely confounding effects of an ongoing (at the time) national multifaceted health education campaign; Working Backs Scotland [14]. Also, 2003 chiropractors were only recruited from members of the British Chiropractic Association, who supplied the sampling frame and postage costs for the chiropractic portion of the survey. Finally, we excluded all 2003 respondents who were not working in clinical practice at the time, as well as those reporting no current clinical caseload of LBP patients.

Data from the 2003 paper questionnaires were converted into electronic format using a pre-coded datafile via SPSS Data Entry software (version 3.0; SPSS Inc., Chicago, IL), which was designed to reduce systematic errors during manual data entry. These data were independently entered by an administrative member of staff and 20% of all manually entered data were then double-checked by one of the researchers (DWE). Original paper questionnaires and their associated consent forms from the 2003 trial were stored securely for 10 years and were then destroyed, leaving fully anonymised data, in accordance with data protection regulations of the time.

### Recruitment and data collection (2023)

To ensure comparability, we intended to gain a similar sample size in 2023 to that obtained in 2003. No formal a priori sample size calculation was performed for the 2023 survey since the primary aim was to replicate the 2003 sampling strategy to allow comparisons across time points. The 2003 sample was designed to reflect proportions of each professional group in the UK at the time. At the time of the 2023 survey, there were approximately 70,000 physiotherapists [39] (of which at least 10,000 were believed to be musculoskeletal by specialty), 5,500 osteopaths [40], and 3,500 chiropractors [41] registered in the UK. Based on these numbers, by inviting as many clinicians as possible, we expected to recruit proportions of eligible clinicians per professional group similar to those achieved in 2003.

We made no attempt to limit the 2023 sample to those who participated in 2003; the two survey samples were treated as independent, although some overlap of participants was possible. Unlike the personally addressed postal questionnaires used in 2003, all 2023 data were collected electronically. These data were captured and stored within a single, protected electronic database, built using REDCap software (Vanderbilt University Medical Centre, Tennessee, USA) [42], which was installed and maintained on physically secure university servers. Entered data were encrypted within the participant’s web browser (HTTPS protocol) before secure transfer to the REDCap database. A bespoke web link (URL) took interested clinicians to a secure webpage that included approved study information. Once this information was confirmed as read and understood, a respondent could proceed to an online consent form that needed to be completed with all required conditions met before access was provided to the online survey itself.

To recruit national samples of physiotherapists, osteopaths, and chiropractors in 2023, we aimed to reproduce the recruitment approach of 2003 in which we sent personalised invitations directly to clinicians. To implement this, we collected email addresses and first names (where publicly available) of all clinicians listed in public-facing searchable ‘find a clinician’ webpages provided by the following professional organisations: the Chartered Society of Physiotherapy (physiotherapists); the Musculoskeletal Association of Chartered Physiotherapists (physiotherapists); the General Osteopathic Council (osteopaths); the Institute of Osteopathy (osteopaths); and the General Chiropractic Council (chiropractors). We ran checks on our compiled list of clinician email addresses to ensure that none were duplicated, given that some overlap between organisation databases was likely. Using first names (where obtainable) and professional group membership (based on the register from which clinician details were collected), we sent a personalised email invitation to each unique clinician in our final list. In addition to this, we separately posted an open survey invitation (including public URL) on social media platforms and additionally asked several UK-based professional associations, societies, and clinical interest groups to distribute this open invitation as widely as possible.

Once all 2023 survey responses were collected, numbers of respondents that were confirmed eligible, consented, and who completed the online survey were recorded. Reasons for non-participation, exclusion, and any withdrawals were also recorded where available. To align these with our 2003 data for the analyses presented here, we excluded any respondent who reported not having a clinical caseload of LBP patients or who did not work clinically within the UK (or a Crown Dependency).

### Allocation to professional groups

Self-declared professional membership determined the group allocation of clinicians in both surveys. To ensure the independence of each professional group in our analyses, clinicians declaring membership to more than one of the three professions were assigned exclusively to one group in the following order: osteopath, chiropractor, and physiotherapist. This meant that a physiotherapist who was also a chiropractor would be allocated exclusively to the chiropractor group. Likewise, a physiotherapist or chiropractor who was also an osteopath would be allocated exclusively to the osteopath group. Number of years qualified was based on the years qualified in the allocated professional group only.

### Survey contents

All 2003 and 2023 participants were asked to provide demographic details (age, gender, etc.) as well as details relating to their clinical work (country in which they practised, professional group membership, registration status, years qualified, contact with other healthcare professionals, LBP caseload, and if they saw patients whose care is NHS-funded), and whether they had lived experience of LBP (ever and during the past 12 months).

We wanted to investigate reported clinician behaviour specifically in relation to acute LBP, defined as less than 6 weeks duration, as distinct from persistent (chronic) LBP, which is widely defined as more than 12 weeks duration [11]. The same single case vignette, describing a patient with acute ‘non-specific’ LBP attending a clinic for the first time (Table 1), was used in both surveys. This was an anglicised version of an expert-derived vignette that has been used to present a case of acute non-specific LBP in several previous clinician studies across different countries [43–46]. Questions relating to this vignette in the two surveys were identical and were designed to capture snapshots of reported clinical behaviour. In both surveys, we informed clinicians that there were “no wrong or right answers” since we wanted to prevent clinicians feeling that this was a test and to avoid any potential response bias that might arise.

**Table 1:** Vignette of acute non-specific LBP patient with no ‘red flags’

A 28 year old woman has been suffering from her first episode of low back pain since lifting a 10 kg box at work three weeks ago
She says that she has been unable to do her job managing a hospital cafeteria for this time, and has not worked since her symptoms began. While anxious to return to work, she feels immobilised by the pain. Her work duties are varied, but generally involve very few physical tasks. She works full-time during the day and has no dependents at home
In terms of activities, she says that she can sit for about 10 min and walk approximately 100 m before she feels she has to stop due to her back pain. She reports that she is able to sleep through the night; however, her back is stiff in the morning and the stiffness lasts for about 10 min. There is no history of trauma or serious illness. The pain is limited to the low back area, without radiation
On physical examination, there is marked limitation of anterior flexion and tenderness in the left paraspinal region. The neurological examination is normal, and her pain does not worsen in response to straight leg raising beyond 90 degrees. All other case history and physical examination findings are unremarkable and she has not previously been seen by any healthcare practitioner since her symptoms began

Multiple-choice questions relating to the vignette (Box 1) covered two categories of clinical behaviour: 1) *investigations* that the clinician would request at this patient’s first visit; and 2) *interventions* that the clinician would choose to use at this first visit. In either category, clinicians could select as many or as few (‘yes’) response options as they liked, including none. All investigation and intervention response options from the 2003 survey were included in the 2023 survey, along with some additions that will be reported separately elsewhere. Here, we will only report responses to options recorded in both surveys to allow for a direct temporal comparison. The wording of questions and response options is provided in the Supplement (Table S1).

Three questions that related to the vignette patient were used to measure the use of recommendations regarding: (i) *activity*, (ii) *work*, and (iii) *bed-rest*. The format used to capture responses to each of these questions was a 5-point ordinal scale [38] (see Table S2 in the Supplement for question wording). In 2003, responses were dichotomised into either ‘guideline-inconsistent’ or ‘guideline-consistent’, based on expert interpretations of guideline recommendations at the time [38]. In this analysis, for both 2003 and 2023 survey responses, we utilised the entire 5-point ordinal scale of each variable and therefore did not dichotomise them.

### Statistical analysis

Data were analysed with R statistical software (version 4.5.0) [60], within the RStudio environment (version 2024.12.1 + 563). Plots were created with the ‘ggplot2’ package for R [47]. Assumptions underlying statistical models were checked before their implementation. Where possible, *p*-values were produced along with test summaries and 95% confidence intervals (CIs) were calculated. Alpha was set at 0.05. Benjamini–Hochberg corrections were applied to all analyses that were repeated across the three professional groups to control for Type I errors.

Kruskal–Wallis H tests were used to evaluate between-group differences of ordinal variables at each time-point (2003 and 2023). Post-hoc pairwise between-group tests of ordinal variables were performed using Dunn’s test. Ordinal regression (proportional odds logistic regression via the ‘MASS’ package for R [48]) was used to estimate within-group changes between all categories of ordinal scales over time (i.e., 2023 values compared to 2003 as reference); odds ratios were obtained by exponentiating logit regression coefficients. In the *activity*, *work*, and *bed-rest* scales, the most restrictive recommendation was used as the reference category in each ordinal model; thus, positive logit coefficients (and corresponding odds ratios > 1) indicated a temporal shift towards less restrictive recommendations.

Chi-square tests were used to test for an overall between-group difference at each time-point (2003 and 2023) in proportions of binary variable responses (‘yes’ versus empty responses in vignette ‘intervention’ and ‘investigation’ response options). Fisher’s Exact Test was used to assess for pairwise between-group differences in a post-hoc manner. Fisher’s Exact Test was also used to compare within-group proportions of responses to each binary variable between 2003 and 2023. Rate ratios were calculated to assess temporal within-group differences in binary variables; values > 1 indicated more frequent selection in 2023 relative to 2003.

All analyses were descriptive and unweighted. We conducted a sensitivity analysis using nearest-neighbour matching without replacement; members of the smallest professional group at each time point were matched to respondents from the other professional groups based on gender, age, and years qualified.

The 2003 sample size was originally determined by the requirements of the RCT [38]. Here, for both cross-sectional inter-professional comparisons and temporal within-group changes, we defined a ‘substantial’ difference as 10 percentage points. A sample size of 389 participants per professional group per survey was required to detect a 10-point percentage difference in the proportion of a binary variable between any two professional groups, with 80% power at a two-sided significance level of 0.05. This calculation was based on a Chi-square test of proportions for two independent groups assuming a baseline proportion of 50% to provide maximum variance and therefore the most conservative estimate. Thus, each survey required a minimum total sample size of 1,167, assuming equal numbers of respondents from each professional group.

### Presentation of results

To retain neutrality in our presentation of results, we followed the categorisation and labelling of interventions for acute LBP used in the 2018 *Lancet Series* on low back pain [11], which drew upon international expert opinion and a synthesis of multiple national guidelines. These categories are: interventions deemed “first-line” for clinicians to “consider for routine use” for acute LBP, which include advice to remain active (*activity*), advice to remain at work (*work*), and advice to avoid bed rest (*bed-rest*); “second-line” or “adjunctive” interventions for acute LBP, which include spinal manipulation, spinal mobilization, massage, and acupuncture; interventions deemed to be of “limited use in selected (acute LBP) patients”, which include exercise therapy (both general exercise and specific exercises) and cognitive behavioural therapy; and other interventions, including those for which there is “insufficient evidence” to make a strong recommendation for acute LBP [11]. Importantly, these categories are only applicable in the context of acute LBP, as defined previously (< 6 weeks duration); the classification of interventions for chronic (persistent) LBP, defined as > 12 weeks duration, would be different [11].

## Results

### Recruitment

As reported previously [37], the direct postal invitation approach of 2003 was open from November 2003 to March 2004 and yielded a response rate of 56% (2,007/3,604). After exclusions, 49% of those approached (1,758/3,604) met all eligibility criteria and provided consent to participate in the study. The numbers of 2003 clinicians allocated to each professional group were: 834 physiotherapists; 592 osteopaths; and 332 chiropractors. Of these, there was one osteopath who was also a chiropractor, four chiropractors who were also physiotherapists, and nine osteopaths who were also physiotherapists. For the smallest group in 2003 (chiropractors, *n* = 332), we had 80% power to detect an inter-professional difference in binary variables of 10.9 percentage points or greater.

The 2023 survey opened in November 2023 and ran until April 2024. In total, 12,826 email invitations were sent directly to registrants of the professional organisations previously described. From these directly emailed invitations, 1,186 clinicians (9%) consented to participate. A further 267 clinicians were recruited via the distributed open invitation, yielding a total of 1,453 clinicians taking part in the 2023 survey. Of these, 1,434 confirmed that their current clinical caseload included LBP patients, and 1,393 stated that they worked in the UK or a Crown Dependency. Hence, 1,388 clinicians were included in the analyses reported here. The numbers of 2023 clinicians allocated to each professional group were: 511 physiotherapists; 621 osteopaths; and 255 chiropractors. Of these, there were two chiropractors who were also physiotherapists; six osteopaths who were also physiotherapists; and one osteopath who was also a chiropractor. For the smallest group in 2023 (chiropractors, *n* = 255), we had 80% power to detect an inter-professional difference in binary variables of 12.4 percentage points or more.

**Fig. 1:**
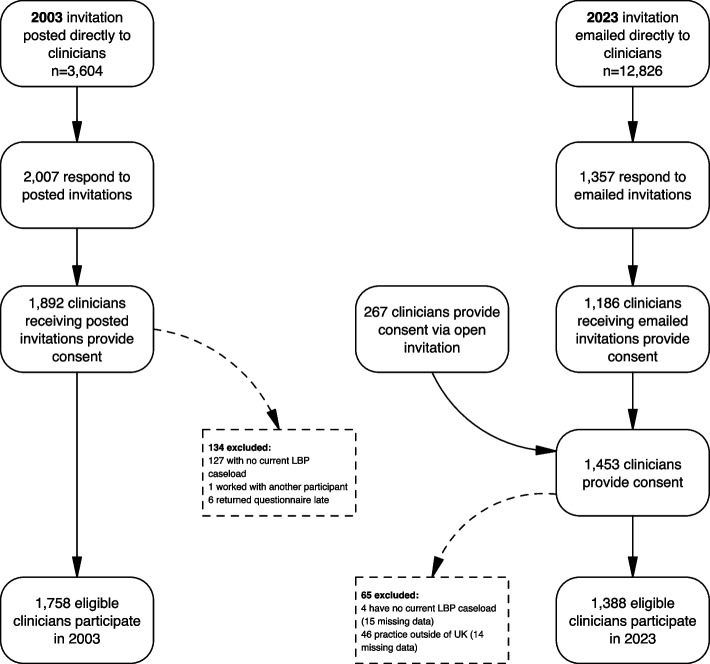
Recruitment to 2003 and 2023 surveys

For temporal (within-group) comparisons between 2003 and 2023, the achieved samples provided 80% power to detect absolute differences of at least 7.9 percentage points for physiotherapists, 8.0 percentage points for osteopaths, and 11.7 percentage points for chiropractors. A summary of recruitment details for both surveys is provided in Fig. 1.

### Participant characteristics

Characteristics of clinicians who took part in each survey, along with temporal comparisons of demographics, are displayed in Table 2. Overall, compared to 2003, in 2023 there was a slightly (but statistically significant) lower proportion of female respondents, participants were an average of 6.2 years older, and they had been qualified an average of 4.0 years longer.

**Table 2:** Characteristics of eligible clinicians responding to 2003 and 2023 surveys

Characteristic	Group	2003	2023	*P*-value
Female gender, n (%)	all	1067/1758 (60.7)	810/1388 (58.4)	< 0.001
	chiropractors	126/332 (37.9)	134/254 (52.7)	< 0.001
	osteopaths	236/591 (39.9)	337/618 (54.5)	0.003
	physiotherapists	705/834 (84.5)	339/509 (66.6)	0.018
Age in years, mean (95% CI)	all	41.4 (40.9, 41.8)	47.6 (47.0, 48.3)	< 0.001
	chiropractors	38.7 (37.6, 39.7)	45.6 (43.9, 47.3)	< 0.001
	osteopaths	41.7 (40.9, 42.5)	48.6 (47.7, 49.6)	< 0.001
	physiotherapists	42.2 (41.6, 42.8)	47.4 (46.4, 48.4)	< 0.001
Years qualified, mean (95% CI)	all	15.3 (14.8, 15.7)	19.3 (18.7, 20.0)	< 0.001
	chiropractors	10.7 (9.87, 11.6)	16.3 (14.9, 17.7)	< 0.001
	osteopaths	12.0 (11.3, 12.7)	17.5 (16.6, 18.5)	< 0.001
	physiotherapists	19.5 (18.9, 20.1)	23.0 (22.0, 24.0)	< 0.001
Lived LBP experience ever, n (%)	all	1403/1758 (79.8)	1159/1388 (83.5)	0.009
	chiropractors	275/332 (82.8)	215/255 (84.3)	0.713
	osteopaths	512/592 (86.4)	541/621 (87.1)	0.811
	physiotherapists	616/834 (73.8)	402/511 (78.7)	0.054
Provides care for NHS patients, n (%)	all	682/1758 (38.8)	220/1388 (15.9)	< 0.001
	chiropractors	31/331 (9.4)	3/255 (1.2)	< 0.001
	osteopaths	71/592 (12.0)	25/596 (4.2)	< 0.001
	physiotherapists	580/834 (69.5)	192/511 (37.6)	< 0.001

In terms of inter-professional comparisons, in both 2003 and 2023 samples, the proportion of females was significantly higher in physiotherapists, compared to chiropractors and osteopaths. However, the female proportion of chiropractor and osteopath respondents increased significantly between 2003 and 2023, whereas the proportion of female physiotherapists decreased significantly over the same period. In both 2003 and 2023, the mean age of chiropractors who responded to the surveys was significantly lower than that of the other two groups. The highest mean age in 2003 was that of physiotherapists, whereas osteopaths had the highest mean age in 2023. Even so, physiotherapists had been qualified significantly longer than any other group in 2003 as well as 2023. Far greater proportions of physiotherapists than any other professional group provided care for NHS patients in both 2003 and 2023; even so, there was a significant reduction in the proportions of each group – including physiotherapists – providing care for NHS patients in the 2023 survey compared to the 2003 survey.

### Investigations

**Fig. 2:**
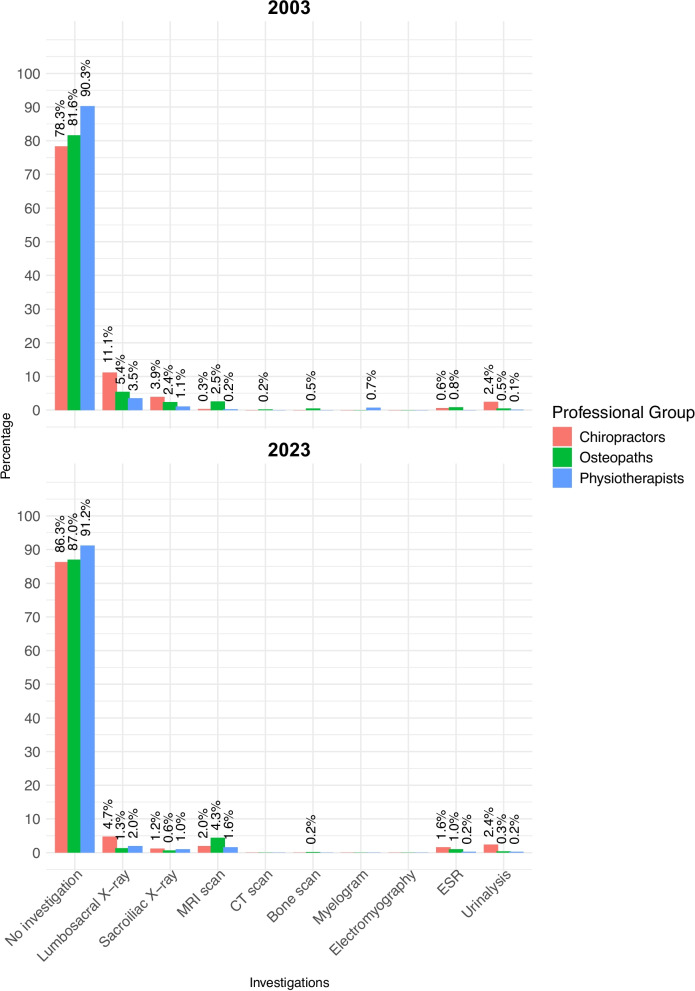
Investigations selected in 2003 and 2023

For the acute LBP patient described in the vignette (Table 1), a large majority of clinicians in all three professional groups explicitly selected ‘no investigations’ in both 2003 (90.3% of physiotherapists, 81.6% of osteopaths, and 78.3% of chiropractors) and 2023 (91.2% of physiotherapists, 87.0% of osteopaths, and 86.3% of chiropractors) (Fig. 2). Over the 20-year period, the proportion opting for no investigations increased significantly in every professional group except physiotherapists (Table 3), who were already the strongest advocates of no investigations in 2003, with a proportion surpassing every other professional group at both time points.

**Table 3:** Most popular investigations and interventions selected in 2003 and 2023

Option	Group	2003 uptake (%)	2023 uptake (%)	Rate ratio (95% CI)	Adjusted *p*-value
*No investigations*	all	1496/1758 (85.1)	1226/1387 (88.39)	1.039 (1.011, 1.068)	0.007
	chiropractors	260/332 (78.31)	220/255 (86.27)	1.102 (1.022, 1.187)	0.02
	osteopaths	483/592 (81.59)	540/621 (86.96)	1.066 (1.015, 1.119)	0.02
	physiotherapists	753/834 (90.29)	466/511 (91.19)	1.01 (0.975, 1.046)	0.63
*Lumbosacral X-ray*	all	98/1758 (5.57)	30/1387 (2.16)	0.388 (0.259, 0.580)	< 0.001
	chiropractors	37/332 (11.1)	12/255 (4.71)	0.422 (0.225, 0.793)	0.009
	osteopaths	32/592 (5.41)	8/621 (1.29)	0.238 (0.111, 0.513)	< 0.001
	physiotherapists	29/834 (3.48)	10/511 (1.96)	0.563 (0.277, 1.145)	0.132
*Spinal* *manipulation*	all	714/1758 (40.61)	446/1387 (32.16)	0.791 (0.719, 0.870)	< 0.001
	chiropractors	298/332 (89.76)*	192/255 (75.29)*	0.839 (0.775, 0.908)	< 0.001
	osteopaths	360/592 (60.81)*	237/621 (38.16)*	0.628 (0.557, 0.707)	< 0.001
	physiotherapists	56/834 (6.71)	17/511 (3.32)	0.495 (0.291, 0.843)	0.009
*Spinal mobilization*	all	901/1758 (51.25)	704/1387 (50.76)	0.99 (0.924, 1.060)	0.774
	chiropractors	55/332 (16.57)*	113/255 (44.31)*	2.675 (2.026, 3.532)	< 0.001
	osteopaths	427/592 (72.13)	420/621 (67.63)	0.938 (0.871, 1.01)	0.091
	physiotherapists	419/834 (50.24)*	171/511 (33.46)*	0.666 (0.579, 0.766)	< 0.001
*Acupuncture*	all	127/1758 (7.22)*	274/1387 (19.75)*	2.533 (2.072, 3.097)	< 0.001
	chiropractors	18/332 (5.42)*	52/255 (20.39)*	3.761 (2.257, 6.267)	< 0.001
	osteopaths	46/592 (7.77)*	135/621 (21.74)*	2.798 (2.042, 3.834)	< 0.001
	physiotherapists	63/834 (7.55)	67/511 (13.11)	1.736 (1.253, 2.404)	0.001
*Massage*	all	926/1758 (52.67)*	924/1387 (66.62)*	1.264 (1.193, 1.339)	< 0.001
	chiropractors	226/332 (68.07)	177/255 (69.41)	1.02 (0.914, 1.138)	0.788
	osteopaths	517/592 (87.33)	509/621 (81.96)	0.939 (0.895, 0.985)	0.016
	physiotherapists	183/834 (21.94)*	238/511 (46.57)*	2.123 (1.812, 2.486)	< 0.001
*Specific exercises*	all	951/1758 (54.1)*	600/1387 (43.26)*	0.799 (0.742, 0.861)	< 0.001
	chiropractors	102/332 (30.72)	66/255 (25.88)	0.842 (0.648, 1.096)	0.231
	osteopaths	265/592 (44.76)	253/621 (40.74)	0.91 (0.799, 1.037)	0.231
	physiotherapists	584/834 (70.02)*	281/511 (54.99)*	0.785 (0.718, 0.859)	< 0.001
*General exercise*	all	715/1758 (40.67)	571/1387 (41.17)	1.011 (0.929, 1.101)	0.798
	chiropractors	103/332 (31.02)	75/255 (29.41)	0.948 (0.739, 1.216)	0.717
	osteopaths	187/592 (31.59)	212/621 (34.14)	1.081 (0.92, 1.27)	0.539
	physiotherapists	425/834 (50.96)	284/511 (55.58)	1.091 (0.985, 1.208)	0.309
*Stretching*	all	705/1758 (40.1)	655/1387 (47.22)	1.177 (1.087, 1.274)	< 0.001
	chiropractors	132/332 (39.76)	112/255 (43.92)	1.105 (0.912, 1.338)	0.312
	osteopaths	431/592 (72.8)	411/621 (66.18)	0.909 (0.844, 0.98)	0.019
	physiotherapists	142/834 (17.03)	132/511 (25.83)	1.517 (1.23, 1.871)	< 0.001
*General advice on back care*	all	1455/1758 (82.76)*	911/1387 (65.68)*	0.793 (0.759, 0.828)	< 0.001
	chiropractors	248/332 (74.7)	178/255 (69.80)	0.934 (0.844, 1.035)	0.193
	osteopaths	449/592 (75.85)	411/621 (66.18)	0.873 (0.812, 0.938)	< 0.001
	physiotherapists	758/834 (90.89)*	322/511 (53.01)*	0.693 (0.647, 0.743)	< 0.001
*Address psychosocial issues*	all	548/1758 (31.17)	380/1387 (27.39)	0.878 (0.787, 0.981)	0.022
	chiropractors	82/332 (24.7)	51/255 (20)	0.81 (0.594, 1.103)	0.196
	osteopaths	118/592 (19.93)	161/621 (25.93)	1.301 (1.055, 1.603)	0.021
	physiotherapists	348/834 (41.73)	168/511 (32.88)	0.788 (0.68, 0.913)	0.004
*Interferential*	all	162/1758 (9.22)	20/1387 (1.44)	0.156 (0.099, 0.247)	< 0.001
	chiropractors	28/332 (8.43)	4/255 (1.57)	0.186 (0.066, 0.524)	< 0.001
	osteopaths	40/592 (6.76)	7/621 (1.13)	0.167 (0.075, 0.369)	< 0.001
	physiotherapists	94/834 (11.27)	9/511 (1.76)	0.156 (0.08, 0.307)	< 0.001
*Ultrasound*	all	231/1758 (13.14)	62/1387 (4.47)	0.34 (0.259, 0.446)	< 0.001
	chiropractors	48/332 (14.46)*	9/255 (3.53)*	0.244 (0.122, 0.488)	< 0.001
	osteopaths	60/592 (10.14)	26/621 (4.19)	0.413 (0.264, 0.645)	< 0.001
	physiotherapists	123/834 (14.75)	27/511 (5.28)	0.358 (0.24, 0.535)	< 0.001

Rate ratios > 1 indicate more frequent selection in 2023 relative to 2003

^*^ ‘Substantial’ (≥ 10%) absolute difference between 2003 and 2023

In 2003, there were significant inter-professional differences in the selection of lumbosacral X-ray, sacroiliac X-ray, MRI scan, and ESR related to the vignette (see Table S3 in the Supplement for full details). In 2023, only lumbosacral X-ray and MRI were significantly different between professional groups. Specifically, lumbosacral X-ray was selected by a greater proportion of chiropractors than any other professional group in both 2003 (11.1%, versus 3.5% of physiotherapists and 5.4% of osteopaths) and 2023 (4.7%, versus 2.0% of physiotherapists and 1.3% of osteopaths). For this LBP scenario, the popularity of lumbar X-ray amongst chiropractors reduced significantly between 2003 and 2023 (Table 3).

Across all participants, MRI scans were slightly (but statistically significantly) more popular in 2023 (2.9%) than in 2003 (1.0%). Indeed, the proportion of each professional group selecting MRI scans had increased in 2023 compared to 2003, although these within-group temporal increases only reached statistical significance in physiotherapists. Between professional groups, MRI scans were significantly more popular with osteopaths than with chiropractors in 2003 (2.5% versus 0.3%) and significantly more popular with osteopaths than with physiotherapists in both 2003 (2.5% versus 0.2%) and 2023 (4.3% versus 1.6%).

### First-line interventions

**Fig. 3:**
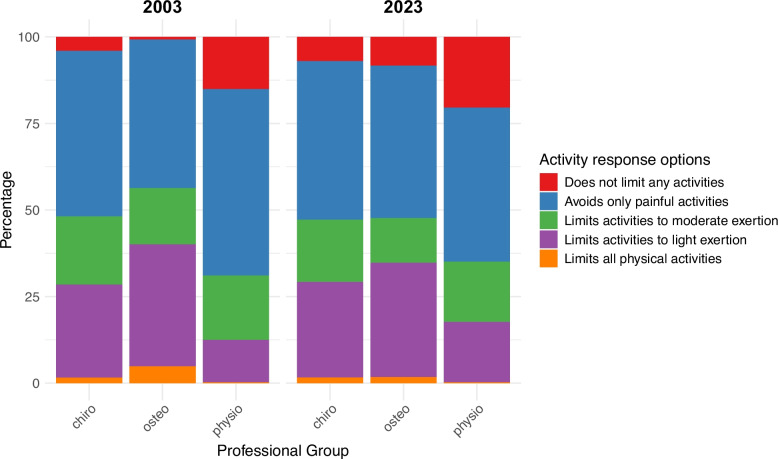
*Activity* recommendations selected in 2003 and 2023

Proportions of ordinal *activity* recommendations relating to the acute LBP vignette for both 2003 and 2023 are displayed in Fig. 3. There was an overall significant difference in *activity* recommendations between professional groups in both 2003 and 2023 (see Table S3 in the Supplement for full details).

Viewed across all participants, there was no significant temporal change in these activity recommendations between 2003 and 2023. However, there were significant inter-professional differences at each time point (see Table S7 in the Supplement for full details). In both 2003 and 2023, physiotherapists provided significantly more active (i.e., less restrictive) *activity* recommendations, compared to the other professional groups. In 2003, osteopaths provided significantly more restrictive *activity* recommendations than any other professional group. Notably, Table 4 shows that osteopaths were the only professional group to show a significant temporal change (towards less restrictive *activity* recommendations) between 2003 and 2023, resulting in there being no significant difference between osteopaths and chiropractors by 2023.

**Table 4:** Temporal comparisons of *work*, *activity,* and *bed-rest* recommendations between 2003 and 2023

Intervention	Group	Coefficient (95% CI)	Odds ratio (95% CI)	Adjusted *p*-value
*Activity*	all	0.046 (−0.087, 0.179)	1.047 (0.917, 1.196)	0.495
	chiropractors	0.063 (−0.245, 0.372)	1.065 (0.782, 1.451)	0.791
	osteopaths	0.428 (0.215, 0.642)	1.535 (1.24, 1.9)	< 0.001
	physiotherapists	−0.029 (−0.241, 0.183)	0.972 (0.786, 1.201)	0.791
*Work*	all	0.135 (0.003, 0.266)	1.144 (1.003, 1.305)	0.066
	chiropractors	0.242 (−0.066, 0.551)	1.274 (0.936, 1.735)	0.124
	osteopaths	0.806 (0.59, 1.023)	2.239 (1.804, 2.78)	< 0.001
	physiotherapists	−0.295 (−0.501, −0.089)	0.745 (0.606, 0.915)	0.007
*Bed-rest*	all	−0.295 (−0.429, −0.161)	0.744 (0.651, 0.851)	< 0.001
	chiropractors	−0.443 (−0.765, −0.121)	0.642 (0.465, 0.886)	0.007
	osteopaths	0.467 (0.249, 0.684)	1.594 (1.283, 1.981)	< 0.001
	physiotherapists	−0.667 (−0.882, −0.452)	0.513 (0.414, 0.636)	< 0.001

The most restrictive recommendation within each ordinal scale was used as the reference category in its respective ordinal regression model. Accordingly, positive coefficients (yielding odds ratios > 1) indicate a temporal shift towards less restrictive recommendations, while negative coefficients (odds ratios < 1) indicate a shift towards more restrictive recommendations

**Fig. 4:**
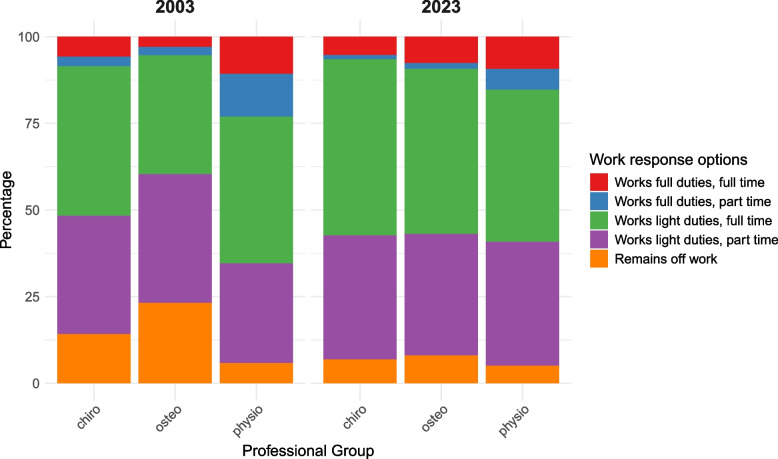
*Work* recommendations selected in 2003 and 2023

Proportions of ordinal *work* recommendations related to the vignette are displayed in Fig. 4. There was a significant difference in *work* recommendations between professional groups in 2003 only (Table S7 in the Supplement), whereby pairwise comparisons showed that physiotherapists provided more active (i.e., less restrictive) *work* recommendations compared to any other professional group. In turn, chiropractors provided less restrictive *work* recommendations than osteopaths in 2003.

By comparison, in 2023 there was no overall significant difference in *work* recommendations between professional groups (Table S7 in the Supplement). Table 4 shows that this temporal change was not only due to osteopaths providing less restrictive *work* recommendations in 2023 compared to 2003, but also because physiotherapists provided significantly more restrictive *work* recommendations in 2023 compared to 2003. Although chiropractors also shifted towards less restrictive *work* recommendations between 2003 and 2023, this did not reach statistical significance.

**Fig. 5:**
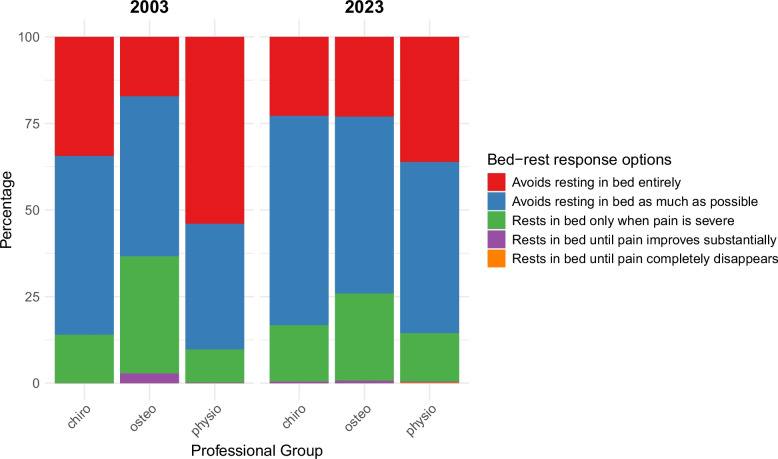
*Bed-*rest recommendations selected in 2003 and 2023

Proportions of ordinal *bed-rest* recommendations related to the vignette are displayed in Fig. 5. There was a significant difference between professional groups in both 2003 and 2023. At both time points, physiotherapists provided more active (i.e., less restrictive) *bed-rest* recommendations compared to any other professional group. Nevertheless, Table 4 shows that physiotherapists provided significantly more restrictive *bed-rest* recommendations in 2023 compared to 2003.

In 2003, osteopaths were more likely to provide more restrictive *bed-rest* recommendations than chiropractors. By 2023 there were no significant differences in *bed-rest* recommendations between osteopaths and chiropractors. This change was due to a combination of osteopaths providing significantly less restrictive *bed-rest* recommendations, and chiropractors providing significantly more restrictive *bed-rest* recommendations, in 2023 compared to 2003 (Table 4).

### Second-line (adjunctive) interventions

**Fig. 6:**
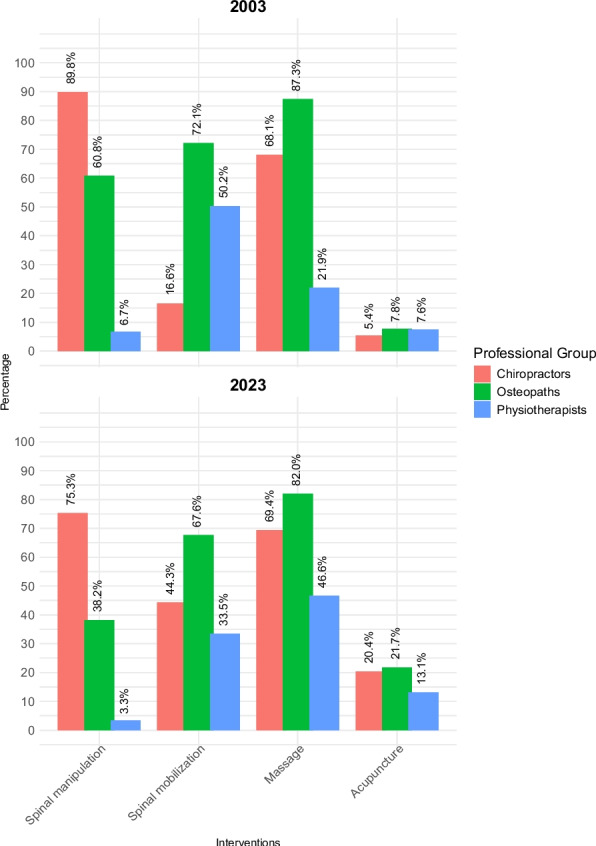
Second-line (adjunctive) interventions selected in 2003 and 2023

In 2003, there was a significant inter-professional difference in every *second-line (adjunctive)* intervention, apart from acupuncture (Table S7 in the Supplement). By comparison, in 2023, there were significant inter-professional differences in all second-line interventions, *including* acupuncture. Figure 6 displays the proportion of each professional group that opted for second-line interventions in 2003 and separately in 2023.

Preferences for second-line interventions within professional groups showed some consistency at both time points. For example, spinal manipulation was the most popular second-line intervention amongst chiropractors in both 2003 (89.8%) and 2023 (75.3%), but the least popular amongst physiotherapists in both 2003 (6.7%) and 2023 (3.3%). On the other hand, massage was the most popular second-line intervention amongst osteopaths in both 2003 (82.0%) and 2023 (87.3%). However, whilst spinal mobilization was the most popular second-line intervention amongst physiotherapists in 2003 (50.2%), the most popular in 2023 was massage (46.6%).

Significant temporal trends in second-line interventions occurred across all three professional groups (Table 3). For example, a significantly smaller proportion of all three professional groups chose spinal manipulation in 2023 compared to 2003: chiropractors, 75.3% versus 89.8%; osteopaths, 38.2% versus 60.8%; and physiotherapists, 3.3% versus 6.7%. By contrast, significantly larger proportions of all three professional groups chose acupuncture in 2023 compared to 2003: chiropractors, 20.4% versus 5.4%; osteopaths, 21.7% versus 7.8%; and physiotherapists, 13.1% versus 7.6%.

Other significant temporal changes in second-line interventions included: more than double the proportion of physiotherapists choosing massage in 2023 (46.6%) compared to 2003 (21.9%); the proportion of chiropractors choosing spinal mobilization in 2023 (16.6%) being less than half of the proportion observed in 2003 (39.8%); and a much lower proportion of osteopaths choosing spinal manipulation in 2023 (38.2%) compared to 2003 (60.8%).

### Limited use interventions

**Fig. 7:**
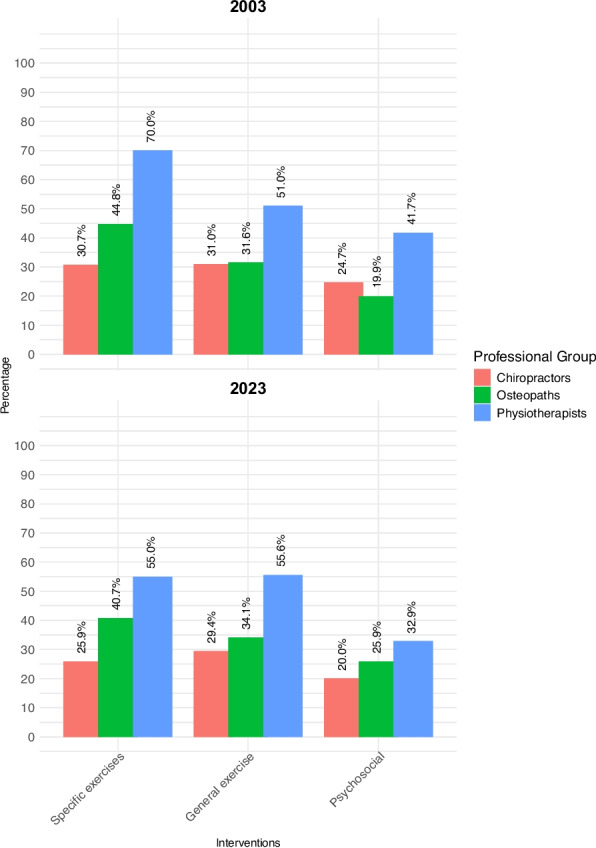
Limited use interventions selected in 2003 and 2023

Figure 7 displays proportions of each professional group that opted for *limited use* interventions in 2003 and 2023. Significant inter-professional differences in these interventions existed in both 2003 and 2023 (see Table S7 in the Supplement). Some inter-professional patterns of *limited use* interventions were consistent across both time points. For example, in both surveys a significantly larger proportion of physiotherapists selected general exercise and specific exercises than any other professional group. Additionally, specific exercises were significantly most popular with physiotherapists and least popular with chiropractors at both time points.

There was an overall significant reduction in the popularity of specific exercises in 2023 compared to 2003 (Table 3), which was driven primarily by physiotherapists. A significantly smaller proportion of physiotherapists (32.9% versus 41.7%) elected to address psychosocial issues in 2023, compared to 2003, whereas the reverse trend was seen in osteopaths (from 19.9% to 25.9%).

### Other interventions

**Fig. 8:**
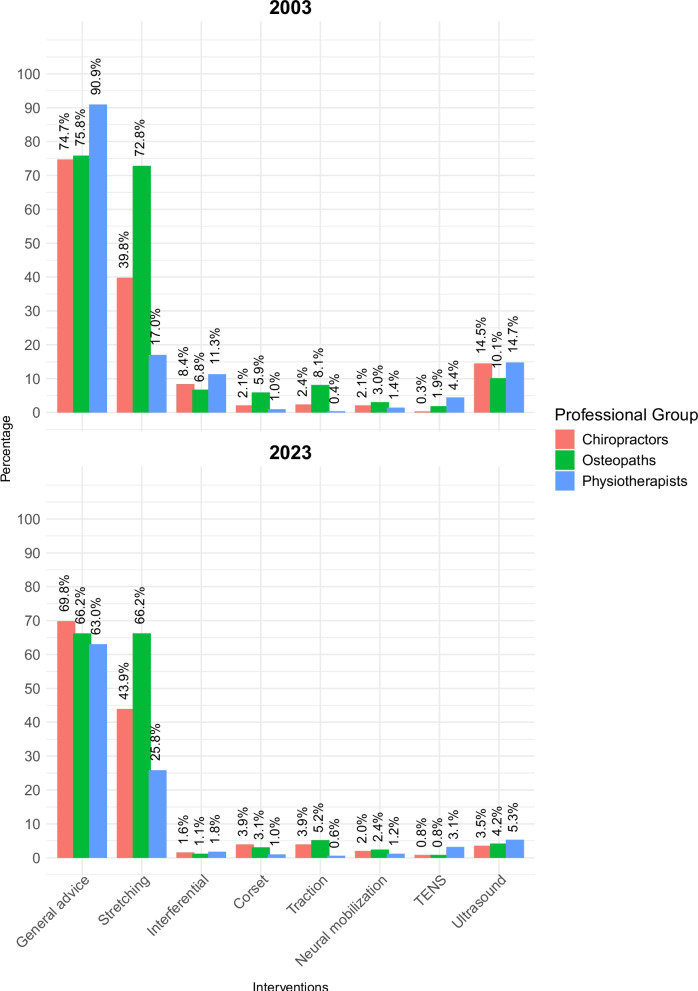
Other interventions selected in 2003 and 2023

*“General advice on back care (lifting, posture, *etc*.)*” was the single most popular remaining intervention with all three professional groups in both 2003 and 2023 (Fig. 8). Despite its cross-group popularity, smaller proportions of each professional group selected general advice in 2023 compared to 2003. Indeed, in 2003 this was significantly more popular with physiotherapists (90.9%) than either chiropractors (74.7%) or osteopaths (75.8%). However, by 2023 general advice had become slightly more popular amongst chiropractors (69.8%) and osteopaths (66.2%) than physiotherapists (63.0%), resulting in inter-professional differences being no longer statistically significant at that time.

In both surveys, a significantly larger proportion of physiotherapists selected to “*address psychosocial issues*” than any other professional group. Stretching was significantly most popular with osteopaths and least popular with physiotherapists at both time points. Although the selection of electrotherapy interventions (interferential therapy, ultrasound, and transcutaneous electrical nerve stimulation [TENS]) was already relatively infrequent in 2003 (Fig. 8), a significant further reduction in the selection of interferential therapy and ultrasound was seen by 2023 within all professional groups (Table 3).

### Substantial changes

Since many findings were statistically significant, which was unsurprising given the large sample sizes, a summary of substantial and statistically significant temporal changes between 2003 and 2023 is displayed in Table 5. Sensitivity analyses using matched samples (gender, age, and years qualified) yielded results consistent with the primary, unadjusted analyses of the full dataset (see Table S8 in Supplement for matched clinician characteristics).

**Table 5:** Substantial temporal changes between 2003 and 2023

Selection	Temporal change
*First-line interventions*	• Physiotherapists shifted towards more restrictive recommendations for *work*, and *bed-rest* • Chiropractors shifted towards more restrictive recommendations for *bed-rest* • Osteopaths shifted towards less restrictive recommendations for *activity*, *work*, and *bed-rest*
*Second-line interventions*	• Chiropractors and osteopaths shifted towards *acupuncture* • Physiotherapists shifted towards *massage* • Chiropractors and osteopaths shifted away from *spinal manipulation* • Chiropractors shifted towards *spinal mobilization* • Physiotherapists shifted away from *spinal mobilization*
*Limited use interventions*	• Physiotherapists shifted away from *specific exercises*
*Other interventions*	• Physiotherapists shifted away from *general advice on back care* • Chiropractors shifted away from *ultrasound*

All temporal changes relate to the acute LBP vignette. For binary variables, ‘substantial’ was defined as an absolute difference of 10% or greater. For ordinal variables (first-line interventions), all statistically significant changes were included

## Discussion

This study is both unique and important because it provides a rigorous ‘generational’ comparison of three professional groups who collectively provide care for a large proportion of individuals seeking care for LBP in the UK [49–51]. Two surveys with identical questions related to the same vignette, large sample sizes, administered two decades apart offered a fascinating insight into how the reported clinical behaviour of these clinicians has evolved over time. In line with our aims, we found significant and substantial temporal changes over the 20-year interval within all three professional groups. We also found significant inter-professional differences in both 2003 and 2023, with several of these differences appearing to reduce over time.

Most clinicians in each of the three professional groups selected ‘no investigations’ for the patient described in the vignette in both 2003 and 2023, with proportions rising over the 20-year period. This general shift away from selecting any investigations is in line with current UK guideline recommendations [26] and international opinion [11]. In particular, the routine use of imaging contrasts with current guidance for LBP management in most countries, including the UK [3, 26]. In line with previous studies [17], proportionally more chiropractors selected to use lumbar spine X-rays than any other professional group in both 2003 and 2023. However, chiropractors significantly reduced this trend by 6.4 percentage-points between 2003 and 2023 (from 37/332 to 12/255); this change could be due to an increased awareness of guideline recommendations and/or targeted regulatory pressure in the UK to reduce the use of ionising radiation [52–54].

Of the *first-line interventions* of *activity*, *work*, and *bed-rest*, a significant overall temporal change was only seen for *bed-rest*. Surprisingly, this overall change was towards more restrictive *bed-rest* recommendations in 2023 compared to 2003, which was driven by the selections of chiropractors and physiotherapists. It is important to note here that, in both 2003 and 2023, physiotherapists selected the least restrictive *activity*, *work* and *bed-rest* recommendations of any professional group. Even so, between 2003 and 2023, physiotherapists exhibited a significant shift towards more restrictive *work* and *bed-rest* recommendations; the underlying reasons for this are currently unknown but could, at least in part, be explained by UK guidelines for LBP not including an explicit recommendation on *bed-rest* since 1999 [24]. Nevertheless, guideline recommendations cannot account for physiotherapists’ corresponding shift towards more restrictive *work* recommendations given that every national LBP guideline published in the UK since 1996 has explicitly covered work-related advice [24–26].

In 2003, osteopaths selected significantly more restrictive recommendations for *activity*, *work*, and *bed-rest* than both chiropractors and physiotherapists. This inter-professional difference may have resulted from a variety of sources, including different beliefs relating to the causes of pain, such as tissue damage, amongst osteopaths, or distinctive perceptions of illness compared to their peers [55–58]. Nevertheless, by 2023, osteopaths’ recommendations for *activity*, *work*, and *bed-rest* were significantly less restrictive, and had become more closely aligned with those of chiropractors. This temporal convergence suggests that osteopaths adopted less restrictive recommendations later than the other professional groups. The reasons for this apparent delay are unclear but may relate to a potentially slower diffusion of research evidence and clinical guidelines amongst osteopaths [59], changes in osteopathy training programmes [60], or broader shifts in osteopaths’ professional beliefs and/or attitudes towards research evidence [18, 55, 59, 61–64].

For *second-line (adjunctive) interventions*, the cross-group usage of spinal manipulation and spinal mobilization decreased significantly between 2003 and 2023, while overall uptake of massage and acupuncture increased significantly. Inter-professional comparisons showed that a greater proportion of chiropractors selected spinal manipulation than any other professional group in both 2003 and 2023, while physiotherapists selected this intervention the least at both time points. This inter-professional pattern is in line with the results of other studies [65, 66]. Moreover, we saw a significant temporal reduction in the popularity of spinal manipulation within all three professional groups over the 20-year period: a reduction of 14.5 percentage points in chiropractors (from 298/332 to 192/255); 22.6 percentage points in osteopaths (from 360/592 to 237/621); and 3.4 percentage points in physiotherapists (from 56/834 to 17/511). This trend is at odds with the recommendations of every national LBP guideline ever published in the UK [24–26], and the majority of national guidelines elsewhere [1–3], which have consistently recommended spinal manipulation as an effective adjunctive intervention for acute LBP. This may instead be a consequence of an increasingly high-profile discourse surrounding the perceived risks of spinal manipulation, even though this is largely focused upon the neck [67–72]. The same perceptions of risk may have also driven the marked temporal shifts observed with the selection of spinal mobilization: a 27.7 percentage point increase in chiropractors choosing spinal mobilization in 2023 (113/255) compared to 2003 (55/332), contrasted with a substantial 16.8 percentage point decrease among physiotherapists (from 419/834 to 171/511) and a modest (but still statistically significant) 4.5 percentage point decrease amongst osteopaths (427/592 to 420/621). Recent commentaries that ‘hands-on’ therapies are declining in popularity within the physiotherapy profession [73–75] are supported by these findings. However, contrary to these claims, we saw a large increase of 24.6 percentage points (from 183/834 to 238/511) in the proportion of physiotherapists selecting massage in 2023 compared to 2003. This contrasted with a modest (but statistically significant) decrease of 5.4 percentage points in osteopaths selecting massage (from 517/592 to 509/621) and an insignificant increase of 1.34 percentage points in chiropractors doing so (from 226/332 to 177/255). Massage was explicitly recommended in the current (2016) NICE guideline for LBP [26]. However, the reason for physiotherapists’ disproportionally large uptake in massage during this period remains unclear, but could stem from continued public demand for hands-on treatment [76–79] combined with the aforementioned migration away from other hands-on interventions that may be perceived as higher risk. Taken together, these temporal changes appear to reflect a broader migration within clinicians’ selections of hands-on interventions over time, progressing from spinal manipulation to spinal mobilization and finally to massage, with physiotherapists at the forefront of this shift.

There was also an increased uptake of acupuncture within all three professions over the two-decade period: a 15.0 percentage point increase from chiropractors (from 18/332 to 52/255); a 14.0 percentage point increase from osteopaths (from 46/592 to 135/621); and a 5.6 percentage point increase from physiotherapists (from 63/834 to 67/511). This trend currently remains unexplained since recommendations surrounding the use of acupuncture in the UK’s NICE guidelines for the management of LBP [25, 26] have been inconsistent over the past 20 years, with a recommendation against the use of acupuncture in the most recent iteration of 2016 but a recommendation for its use in the 2009 guideline. However, a more recent recommendation favouring the use of acupuncture, within the 2021 NICE guideline for the management of chronic pain [80], could have been influential by 2023. Additionally, current LBP guidelines from several other countries recommend the use of acupuncture for acute LBP management [81] and may have exerted influence beyond their formal jurisdiction.

Regarding *limited use interventions* [11], there was a significant overall decrease in the selection of specific exercises for the acute LBP vignette between 2003 and 2023, driven primarily by physiotherapists: the proportion of physiotherapists reduced by 15.03% (from 584/834 to 281/511); the proportion of chiropractors reduced by 4.84% (from 102/332 to 66/255); while the proportion of osteopaths reduced by 4.02% (from 265/592 to 253/621). This migration away from specific exercises may reflect uncertainty arising from clinical trial results indicating equipoise between different types of specific exercises for acute LBP [82]. However, physiotherapists also migrated away from providing general advice on back care (from 90.9% [758/834] to 53.0% [322/511]), and away from addressing psychosocial issues (from 41.7% [348/834] to 32.9% [168/511]), suggesting that they are not merely transitioning towards a hands-off approach [73–75], and that a more complex shift in practice is taking place. Similarly, there was an overall shift away from the use of electrotherapy interventions, particularly interferential therapy and ultrasound, which was again led by physiotherapists.

A strength of this study is the large sample sizes achieved in both 2003 (*n* = 1,758) and 2023 (*n* = 1,388) surveys. Although the number of chiropractors was lower than that of other groups at each time point, this is in line with the size of the professional groups in the UK, and sufficient power was still achieved to detect substantial differences involving this group. Reassuringly, findings from the sensitivity analyses were aligned with those from the full dataset, supporting the robustness of our results.

Another strength was the uniformity in our approach to data collection in both surveys, with most participants being recruited via personally addressed direct contact invitations. However, a potential weakness results from the relatively low response rate of 9.2% (1,186/12,826) from the 2023 directly emailed survey invitations, compared to the 55.7% (2,007/3,604) response rate achieved using directly posted questionnaires in 2003. A lower response rate was expected from unsolicited email invitations to an online survey [83], especially from busy clinicians [84, 85], but could nonetheless have resulted in some response bias. The demographics of respondents were largely similar between the two surveys but having fewer respondents caring for NHS patients in 2023 compared to 2003 may have contributed to some of the trends we have described [86]. Another potential limitation of this study is that we used a clinical vignette to capture reported behaviour, rather than observing actual clinician behaviour. However, vignettes have not only been shown to be reliable, stable and valid measures of practitioner behaviour; they are also more accurate than patient case notes (chart abstraction), which are often used as a measure of practitioner behaviour [87–90]. Directly observing the behaviour of thousands of clinicians would also be prohibitively time-consuming and expensive, whereas vignettes ensure that all clinicians assess the same patient scenario, making fair inter-professional comparisons feasible in a way that would be unworkable with real patients. We deliberately utilised an expert-derived vignette scenario that described a young person with uncomplicated ‘non-specific’ LBP, presenting with no ‘red flags’, which has been used in several previous studies to elicit clinician beliefs and reported behaviour. Whilst this approach provided standardisation, we were unable to assess reported behaviour in response to chronic non-specific LBP, which may have been different and will have provided a more complete picture of temporal trends and inter-professional similarities and differences. Future studies should ideally investigate clinical behaviour with both acute and chronic LBP.

## Conclusions

In both 2003 and 2023, there were significant inter-professional differences in the investigations and interventions reported for an identical acute non-specific LBP scenario by UK physiotherapists, osteopaths, and chiropractors. There were also significant and substantial temporal changes over this 20-year period within each professional group, which differed between groups, and at times diverged from guideline recommendations. Some inter-professional differences appear to be narrowing over time, suggesting partial convergence of practice.

## Supplementary Information

Supplementary Material 1.

Supplementary Material 2.

## Data Availability

Data are provided within the supplementary information files.

## References

[1] Koes BW, van Tulder MW, Ostelo R, Kim Burton A, Waddell G. Clinical guidelines for the management of low back pain in primary care: an international comparison. Spine (Phila Pa 1976). 2001;26(22):2504–13; discussion 2513-2504. 11707719 10.1097/00007632-200111150-00022

[2] Koes BW, van Tulder M, Lin CW, Macedo LG, McAuley J, Maher C. An updated overview of clinical guidelines for the management of non-specific low back pain in primary care. Eur Spine J. 2010;19(12):2075–94. 20602122 10.1007/s00586-010-1502-y PMC2997201

[3] Oliveira CB, Maher CG, Pinto RZ, Traeger AC, Lin CC, Chenot JF, et al. Clinical practice guidelines for the management of non-specific low back pain in primary care: an updated overview. Eur Spine J. 2018;27(11):2791–803. 29971708 10.1007/s00586-018-5673-2

[4] Waddell G, O’Connor M, Boorman S, Torsney B. Working backs Scotland: a public and professional health education campaign for back pain. Spine (Phila Pa 1976). 2007;32(19):2139–43. 17762817 10.1097/BRS.0b013e31814541bc

[5] Buchbinder R. Self-management education en masse: effectiveness of the back pain: don’t take it lying down mass media campaign. Med J Aust. 2008;189(S10):S29-32. 19143582 10.5694/j.1326-5377.2008.tb02207.x

[6] Werner EL, Ihlebaek C, Laerum E, Wormgoor ME, Indahl A. Low back pain media campaign: no effect on sickness behaviour. Patient Educ Couns. 2008;71(2):198–203. 18242932 10.1016/j.pec.2007.12.009

[7] Suman A, Schaafsma FG, Bamarni J, van Tulder MW, Anema JR. A multimedia campaign to improve back beliefs in patients with non-specific low back pain: a process evaluation. BMC Musculoskelet Disord. 2017;18(1):200. 28521761 10.1186/s12891-017-1551-z PMC5437407

[8] Gross DP, Russell AS, Ferrari R, Battie MC, Schopflocher D, Hu R, et al. Evaluation of a Canadian back pain mass media campaign. Spine (Phila Pa 1976). 2010;35(8):906–13. 20308943 10.1097/BRS.0b013e3181c91140

[9] Hoy D, March L, Brooks P, Blyth F, Woolf A, Bain C, et al. The global burden of low back pain: estimates from the global burden of disease 2010 study. Ann Rheum Dis. 2014;73(6):968–74. 24665116 10.1136/annrheumdis-2013-204428

[10] Clark S, Horton R. Low back pain: a major global challenge. Lancet. 2018;391(10137):2302. 29573869 10.1016/S0140-6736(18)30725-6

[11] Foster NE, Anema JR, Cherkin D, Chou R, Cohen SP, Gross DP, et al. Prevention and treatment of low back pain: evidence, challenges, and promising directions. Lancet. 2018;391(10137):2368–83. 29573872 10.1016/S0140-6736(18)30489-6

[12] Hartvigsen J, Hancock MJ, Kongsted A, Louw Q, Ferreira ML, Genevay S, et al. What low back pain is and why we need to pay attention. Lancet. 2018;391(10137):2356–67. 29573870 10.1016/S0140-6736(18)30480-X

[13] Buchbinder R, van Tulder M, Oberg B, Costa LM, Woolf A, Schoene M, et al. Low back pain: a call for action. Lancet. 2018;391(10137):2384–8. 29573871 10.1016/S0140-6736(18)30488-4

[14] Waddell G. The back pain revolution. 2nd ed. Edinburgh: Churchill Livingstone; 2004.

[15] Toloui-Wallace J, Forbes R, Thomson OP, Setchell J. When worlds collide: experiences of physiotherapists, chiropractors, and osteopaths working together. Musculoskelet Sci Pract. 2022;60:102564. 35462317 10.1016/j.msksp.2022.102564

[16] Toloui-Wallace J, Forbes R, Thomson OP, Costa N. Fluid professional boundaries: ethnographic observations of co-located chiropractors, osteopaths and physiotherapists. BMC Health Serv Res. 2024;24(1):344. 38491351 10.1186/s12913-024-10738-1 PMC10943826

[17] Jenkins HJ, Downie AS, Moore CS, French SD. Current evidence for spinal X-ray use in the chiropractic profession: a narrative review. Chiropr Man Therap. 2018;26:48. 30479744 10.1186/s12998-018-0217-8 PMC6247638

[18] Thomson OP, Petty NJ, Moore AP. Diagnostic reasoning in osteopathy – a qualitative study. Int J Osteopath Med. 2014;17(2):83–93.

[19] Sherman KJ, Cherkin DC, Deyo RA, Erro JH, Hrbek A, Davis RB, et al. The diagnosis and treatment of chronic back pain by acupuncturists, chiropractors, and massage therapists. Clin J Pain. 2006;22(3):227–34. 16514321 10.1097/01.ajp.0000169668.62900.ca

[20] Vickers A, Zollman C. ABC of complementary medicine. The manipulative therapies: osteopathy and chiropractic. BMJ. 1999;319(7218):1176–9. 10541511 10.1136/bmj.319.7218.1176 PMC1116959

[21] Clijsters M, Fronzoni F, Jenkins H. Chiropractic treatment approaches for spinal musculoskeletal conditions: a cross-sectional survey. Chiropr Man Therap. 2014;22(1):33. 25309722 10.1186/s12998-014-0033-8 PMC4193988

[22] Walker KR, Swait G. Characterisation of the multimodal care provided by UK chiropractors: a survey. Research Square; 2022.

[23] Royal College of General Practitioners. Clinical guidelines for the managememt of acute low back pain. London: Royal College of General Practitioners; 1996.

[24] Royal College of General Practitioners. Clinical guidelines for the managememt of acute low back pain. London: Royal College of General Practitioners; 1999.

[25] National Institute for Health and Care Excellence (NICE). Low back pain: early management of persistent non-specific low back pain [NICE clinical guideline 88]. London: National Institute for Health and Care Excellence (NICE); 2009.

[26] National Institute for Health and Care Excellence (NICE). Low back pain and sciatica in over 16s: assessment and management [NG59]. London: National Institute for Health and Care Excellence (NICE); 2016. 27929617

[27] Oikonomou E, Carthey J, Macrae C, Vincent C. Patient safety regulation in the NHS: mapping the regulatory landscape of healthcare. BMJ Open. 2019;9(7):e028663. 31289082 10.1136/bmjopen-2018-028663 PMC6615819

[28] Yeung AWK, Kletecka-Pulker M, Klager E, Eibensteiner F, Doppler K, El-Kerdi A, et al. Patient safety and legal regulations: a total-scale analysis of the scientific literature. J Patient Saf. 2022;18(7):e1116–23. 35617635 10.1097/PTS.0000000000001040

[29] McDonald F. Patient safety law: regulatory change in Britain and Canada [PhD thesis]. Schulich School of Law, Dalhousie University; 2010.

[30] Waring J. Adaptive regulation or governmentality: patient safety and the changing regulation of medicine. Sociol Health Illn. 2007;29(2):163–79. 17381811 10.1111/j.1467-9566.2007.00527.x

[31] Karas M, Sheen NJL, North RV, Ryan B, Bullock A. Continuing professional development requirements for UK health professionals: a scoping review. BMJ Open. 2020;10(3):e032781. 32161156 10.1136/bmjopen-2019-032781 PMC7066625

[32] Allied Health Professions Federation. UK Allied Health Professions public health strategic framework 2019–2024. Allied Health Professions Federation; 2019.

[33] Adams TL. Health professional regulation in historical context: Canada, the USA and the UK (19th century to present). Hum Resour Health. 2020;18(1):72. 33076923 10.1186/s12960-020-00501-y PMC7572238

[34] Leslie K, Moore J, Robertson C, Bilton D, Hirschkorn K, Langelier MH, et al. Regulating health professional scopes of practice: comparing institutional arrangements and approaches in the US, Canada, Australia and the UK. Hum Resour Health. 2021;19(1):15. 33509209 10.1186/s12960-020-00550-3 PMC7841037

[35] Leslie K, Nelson S, Deber R, Gilmour J. Policy tensions in regulatory reform: changes to regulation of health professions in Australia, the United Kingdom, and Ontario, Canada. J Nurs Regul. 2018;8(4):32–42.

[36] Allsop J, Jones K. Chapter six: regulating the regulators: the rise of the United Kingdom professional standards authority. In: hamberlain JM, Dent M, Saks M, Allsop J, Jones K, editors. Professional health regulation in the public interest: international perspectives. Policy Press; 2018. p. 93–116.

[37] Evans DW, Breen AC, Pincus T, Sim J, Underwood M, Vogel S, et al. The effectiveness of a posted information package on the beliefs and behavior of musculoskeletal practitioners: the UK chiropractors, osteopaths, and musculoskeletal physiotherapists low back pain management (COMPLeMENT) randomized trial. Spine (Phila Pa 1976). 2010;35(8):858–66. 20308941 10.1097/BRS.0b013e3181d4e04b

[38] Evans DW, Foster NE, Underwood M, Vogel S, Breen AC, Pincus T. Testing the effectiveness of an innovative information package on practitioner reported behaviour and beliefs: the UK chiropractors, osteopaths and musculoskeletal physiotherapists low back pain managemENT (COMPLeMENT) trial [ISRCTN77245761]. BMC Musculoskelet Disord. 2005;6:41. 16033646 10.1186/1471-2474-6-41 PMC1208895

[39] Health and Care Professions Council. Register summary. London: Health and Care Professions Council; 2023.

[40] Professional Standards Authority. Performance review. Periodic review 2023/24. London: Professional Standards Authority; 2024.

[41] General Chiropractic Council. Annual report & accounts 2023. London: General Chiropractic Council; 2023.

[42] Harris PA, Taylor R, Thielke R, Payne J, Gonzalez N, Conde JG. Research electronic data capture (REDCap)–a metadata-driven methodology and workflow process for providing translational research informatics support. J Biomed Inform. 2009;42(2):377–81. 18929686 10.1016/j.jbi.2008.08.010 PMC2700030

[43] Bombardier C, Jansz G, Maetzel A. Primary care physicians’ knowledge, confidence, and attitude in the management of acute low back pain (ALBP). Arthritis Rheum. 1995;38:5385.

[44] Rainville J, Carlson N, Polatin P, Gatchel RJ, Indahl A. Exploration of physicians’ recommendations for activities in chronic low back pain. Spine (Phila Pa 1976). 2000;25(17):2210–20. 10973405 10.1097/00007632-200009010-00012

[45] Buchbinder R, Jolley D, Wyatt M. 2001 Volvo award winner in clinical studies: effects of a media campaign on back pain beliefs and its potential influence on management of low back pain in general practice. Spine (Phila Pa 1976). 2001;26(23):2535–42. 11725233 10.1097/00007632-200112010-00005

[46] Bishop A, Foster NE. Do physical therapists in the United Kingdom recognize psychosocial factors in patients with acute low back pain? Spine (Phila Pa 1976). 2005;30(11):1316–22. 15928559 10.1097/01.brs.0000163883.65321.33

[47] Wickham H. ggplot2: elegant graphics for data analysis. 2nd ed. New York: Springer-Verlag; 2016.

[48] Ripley B, Venables B, Bates DM, Hornik K, Gebhardt A, Firth D, et al. Package ‘mass.’ Cran r. 2013;538:113–20.

[49] Harvey E, Burton AK, Moffett JK, Breen A, team UBt. Spinal manipulation for low-back pain: a treatment package agreed to by the UK chiropractic, osteopathy and physiotherapy professional associations. Man Ther. 2003;8(1):46–51. 12635637 10.1054/math.2002.0472

[50] Breen AC, Carr E, Langworthy JE, Osmond C, Worswick L. Back pain outcomes in primary care following a practice improvement intervention:- a prospective cohort study. BMC Musculoskelet Disord. 2011;12:28. 21272310 10.1186/1471-2474-12-28 PMC3040163

[51] Maniadakis N, Gray A. The economic burden of back pain in the UK. Pain. 2000;84(1):95–103. 10601677 10.1016/S0304-3959(99)00187-6

[52] Health and Safety Executive. Evaluation: ionising radiation targeted inspections, chiropractors. London; 2019.

[53] Royal College of Chiropractors. A statement from the chiropractic professional associations and RCC regarding planned inspections of chiropractors with X-ray facilities. London: General Chiropractic Council; 2018.

[54] Royal College of Chiropractors. Recommendations by the chiropractic professional associations and the Royal College of Chiropractors following the ionising radiation targeted Inspections by the Health and Safety Executive (HSE) January to March 2019. London: General Chiropractic Council; 2019.

[55] Thomson OP, Petty NJ, Moore AP. Osteopaths’ professional views, identities and conceptions–a qualitative grounded theory study. Int J Osteopath Med. 2014;17(3):146–59.

[56] Bar-Zaccay A, Bailey D. The attitudes and beliefs of UK osteopaths towards the management of low back pain: a cross-sectional study. Int J Osteopath Med. 2018;28:42–7.

[57] Macdonald M, Vaucher P, Esteves JE. The beliefs and attitudes of UK registered osteopaths towards chronic pain and the management of chronic pain sufferers-a cross-sectional questionnaire based survey. Int J Osteopath Med. 2018;30:3–11.

[58] Smith K, Thomson OP. What do UK osteopaths view as the safest lifting posture, and how are these views influenced by their back pain beliefs? Int J Osteopath Med. 2020;37:10–6.

[59] Sundberg T, Leach MJ, Thomson OP, Austin P, Fryer G, Adams J. Attitudes, skills and use of evidence-based practice among UK osteopaths: a national cross-sectional survey. BMC Musculoskelet Disord. 2018;19(1):439. 30526551 10.1186/s12891-018-2354-6 PMC6286591

[60] Engel R. The seven drivers of change in osteopathic education. Int J Osteopath Med. 2025;55:100740.

[61] Evans DW, Foster NE, Vogel S, Breen AC. Implementing evidence-based practice in the UK physical therapy professions: do they want it and do they feel they need it? In: International Forum for Low Back Pain Research. Linkoping; 2005.

[62] Leach J. Towards an osteopathic understanding of evidence. Int J Osteopath Med. 2008;11(1):3–6.

[63] Humpage C. Opinions on research and evidence based medicine within the UK osteopathic profession: a thematic analysis of public documents 2003–2009. Int J Osteopath Med. 2011;14(2):48–56.

[64] Weber V, Rajendran D. UK trained osteopaths’ relationship to evidence based practice - an analysis of influencing factors. Int J Osteopath Med. 2018;29:15–25.

[65] Beliveau PJH, Wong JJ, Sutton DA, Simon NB, Bussieres AE, Mior SA, et al. The chiropractic profession: a scoping review of utilization rates, reasons for seeking care, patient profiles, and care provided. Chiropr Man Therap. 2017;25:35. 29201346 10.1186/s12998-017-0165-8 PMC5698931

[66] Hurwitz EL. Epidemiology: spinal manipulation utilization. J Electromyogr Kinesiol. 2012;22(5):648–54. 22289432 10.1016/j.jelekin.2012.01.006

[67] Stevinson C, Ernst E. Risks associated with spinal manipulation. Am J Med. 2002;112(7):566–71. 12015249 10.1016/s0002-9343(02)01068-9

[68] Maiers M, Evans R, Hartvigsen J, Schulz C, Bronfort G. Adverse events among seniors receiving spinal manipulation and exercise in a randomized clinical trial. Man Ther. 2015;20(2):335–41. 25454683 10.1016/j.math.2014.10.003

[69] Hebert JJ, Stomski NJ, French SD, Rubinstein SM. Serious adverse events and spinal manipulative therapy of the low back region: a systematic review of cases. J Manipulative Physiol Ther. 2015;38(9):677–91. 23787298 10.1016/j.jmpt.2013.05.009

[70] Paige NM, Miake-Lye IM, Booth MS, Beroes JM, Mardian AS, Dougherty P, et al. Association of spinal manipulative therapy with clinical benefit and harm for acute low back pain: systematic review and meta-analysis. JAMA. 2017;317(14):1451–60. 28399251 10.1001/jama.2017.3086 PMC5470352

[71] Swait G, Finch R. What are the risks of manual treatment of the spine? A scoping review for clinicians. Chiropr Man Therap. 2017;25:37. 29234493 10.1186/s12998-017-0168-5 PMC5719861

[72] Rushton A, Carlesso LC, Flynn T, Hing WA, Rubinstein SM, Vogel S, et al. International framework for examination of the cervical region for potential of vascular pathologies of the neck prior to musculoskeletal intervention: international IFOMPT cervical framework. J Orthop Sports Phys Ther. 2023;53(1):7–22. 36099171 10.2519/jospt.2022.11147

[73] Jull G, Moore A. Hands on, hands off? The swings in musculoskeletal physiotherapy practice. Man Ther. 2012;17(3):199–200. 22482936 10.1016/j.math.2012.03.009

[74] Reid D, Cook C, Sizer PS, Froment F, Showalter CR, Brismee JM. Is orthopaedic manipulative physical therapy not fashionable anymore? Lessons learned from 2016 IFOMPT meeting and future directions. J Man Manip Ther. 2017;25(1):1–2. 28855786 10.1080/10669817.2017.1272817 PMC5539572

[75] Mintken PE, Rodeghero J, Cleland JA. Manual therapists - have you lost that loving feeling?! J Man Manip Ther. 2018;26(2):53–4. 29686478 10.1080/10669817.2018.1447185 PMC5901426

[76] Pincus T, Vogel S, Savage R, Newman S. Patients’ satisfaction with osteopathic and GP management of low back pain in the same surgery. Complement Ther Med. 2000;8(3):180–6. 11068348 10.1054/ctim.2000.0378

[77] Sharp D, Lorenc A, Morris R, Feder G, Little P, Hollinghurst S, et al. Complementary medicine use, views, and experiences: a national survey in England. BJGP Open. 2018;2(4):bjgpopen18X101614. 30723800 10.3399/bjgpopen18X101614 PMC6348322

[78] Thomas KJ, Nicholl JP, Coleman P. Use and expenditure on complementary medicine in England: a population based survey. Complement Ther Med. 2001;9(1):2–11. 11264963 10.1054/ctim.2000.0407

[79] Westmoreland JL, Williams NH, Wilkinson C, Wood F, Westmoreland A. Should your GP be an osteopath? Patients’ views of an osteopathy clinic based in primary care. Complement Ther Med. 2007;15(2):121–7. 17544863 10.1016/j.ctim.2005.11.006

[80] National Institute for Health and Care Excellence (NICE). Chronic pain (primary and secondary) in over 16s: assessment of all chronic pain and management of chronic primary pain. NICE guideline [NG193]. London: National Institute for Health and Care Excellence (NICE); 2021. 33939353

[81] Zhou T, Salman D, McGregor AH. Recent clinical practice guidelines for the management of low back pain: a global comparison. BMC Musculoskelet Disord. 2024;25(1):344. 38693474 10.1186/s12891-024-07468-0 PMC11061926

[82] Karlsson M, Bergenheim A, Larsson MEH, Nordeman L, van Tulder M, Bernhardsson S. Effects of exercise therapy in patients with acute low back pain: a systematic review of systematic reviews. Syst Rev. 2020;9(1):182. 32795336 10.1186/s13643-020-01412-8 PMC7427286

[83] Wu MJ, Zhao K, Fils-Aime F. Response rates of online surveys in published research: a meta-analysis. Comput Human Behav Rep. 2022;7:100206.

[84] Wiebe ER, Kaczorowski J, MacKay J. Why are response rates in clinician surveys declining? Can Fam Physician. 2012;58(4):e225-228. 22611609 PMC3325475

[85] Flanigan TS, McFarlane E, Cook S. Conducting survey research among physicians and other medical professionals: a review of current literature. In: Proceedings of the survey research methods section, American Statistical Association: 2008. 2008. p. 4136–4147.

[86] Lowson K, Jenks M, Filby A, Carr L, Campbell B, Powell J. Examining the implementation of NICE guidance: cross-sectional survey of the use of NICE interventional procedures guidance by NHS trusts. Implement Sci. 2015;10(1):93. 26122560 10.1186/s13012-015-0283-4 PMC4486420

[87] Peabody JW, Luck J, Glassman P, Dresselhaus TR, Lee M. Comparison of vignettes, standardized patients, and chart abstraction: a prospective validation study of 3 methods for measuring quality. JAMA. 2000;283(13):1715–22. 10755498 10.1001/jama.283.13.1715

[88] Peabody JW, Luck J, Glassman P, Jain S, Hansen J, Spell M, et al. Measuring the quality of physician practice by using clinical vignettes: a prospective validation study. Ann Intern Med. 2004;141(10):771–80. 15545677 10.7326/0003-4819-141-10-200411160-00008

[89] Luck J, Peabody JW, Dresselhaus TR, Lee M, Glassman P. How well does chart abstraction measure quality? A prospective comparison of standardized patients with the medical record. Am J Med. 2000;108(8):642–9. 10856412 10.1016/s0002-9343(00)00363-6

[90] Veloski J, Tai S, Evans AS, Nash DB. Clinical vignette-based surveys: a tool for assessing physician practice variation. Am J Med Qual. 2005;20(3):151–7. 15951521 10.1177/1062860605274520

