# Supplementary material for: The changing face of acute low back pain management by physiotherapists, osteopaths and chiropractors in the UK: a 20-year comparison from 2003 to 2023
Source: BMC Musculoskelet Disord. 2025 Oct 2;26:887. doi: 10.1186/s12891-025-09192-9 (PMC12490145; doi:10.1186/s12891-025-09192-9)
Supplement: Supplementary file 1 — Supplementary Material 1. [file 12891_2025_9192_MOESM1_ESM.docx]

**Supplementary material**

**Table S1. Wording of binary (‘yes’ or empty) response options related to the vignette**

| **Item** | **Wording in questionnaire** |
| --- | --- |
| **Investigation question** | Which investigations would you want to be performed on this patient following this visit? (These are listed in alphabetical order. Please select all that apply) |
| *No investigations* | Would not want any investigations to be performed |
| *Bone scan* | Bone scan |
| *CT scan* | CT scan |
| *EMG* | Electromyography / nerve conduction |
| *ESR* | Erythrocyte sedimentation rate (ESR) |
| *Myelogram* | Myelogram |
| *Urinalysis* | Urinalysis |
| **Intervention question** | What form(s) of care would you give to this patient at this first visit? (These are listed in alphabetical order. Please select all that apply) |
| *Acupuncture* | Acupuncture / dry needling |
| *Massage* | Soft-tissue techniques (e.g. massage) |
| *Spinal manipulation* | Spinal manipulation (‘Adjustment’, ‘High velocity thrust’, or ‘Maitland Grade V’) |
| *Spinal mobilization* | Spinal mobilization (‘Articulation’, ‘Maitland Grade I-IV’, ‘NAGS’, ‘SNAGS’, etc.) |
| *General exercise* | General exercise |
| *Specific exercises* | Specific exercises |
| *Stretching* | Stretching (e.g. active, passive, ‘Muscle Energy’, PNF, etc.) |
| *General advice on back care* | General advice on back care (lifting, posture, etc.) |
| *Address psychosocial issues* | Address psychosocial issues |
| *Neural mobilization* | Neural mobilisation / neurodynamics |
| *Traction* | Mechanical spinal traction |
| *Corset* | Lumbar support, belt or corset |
| *Interferential* | Interferential |
| *TENS* | Transcutaneous Electrical Nerve Stimulation (TENS) |
| *Ultrasound* | Ultrasound |

**Table S2. Wording of ordinal questions and response options related to the vignette**

Response options are ordered (and were presented to participants) with decreasing restriction.

| **Label** | **Header** | **Response option** |
| --- | --- | --- |
| ***Activity*** | Until pain improves, I would recommend that this patient: | Limits all physical activities |
|  |  | Limits activities to light exertion |
|  |  | Limits activities to moderate exertion |
|  |  | Avoids only painful activities |
|  |  | Does not limit any activities |
| ***Work*** | Until pain improves, I would recommend that this patient: | Remains off work |
|  |  | Works light duties, part time |
|  |  | Works light duties, full time |
|  |  | Works full duties, part time |
|  |  | Works full duties, full time |
| ***Bed-rest*** | I would recommend that this patient: | Rests in bed until pain completely disappears |
|  |  | Rests in bed until pain improves substantially |
|  |  | Rests in bed only when pain is severe |
|  |  | Avoids resting in bed as much as possible |
|  |  | Avoids resting in bed entirely |

**Table S3. Overall inter-professional group comparisons in 2003 and 2023**

| **Intervention / investigation** | **Year** | **Test statistic** | ***P*-value** |
| --- | --- | --- | --- |
| ***No investigations*** | 2003 | *X*^2^ = 35.51, *df* = 2 | < 0.001 |
|  | 2023 | *X*^2^ = 6.27, *df* = 2 | 0.043 |
| ***Lumbosacral X-ray*** | 2003 | *X*^2^ = 26.57, *df* = 2 | < 0.001 |
|  | 2023 | *X*^2^ = 10.14, *df* = 2 | 0.006 |
| ***Sacroiliac X-ray*** | 2003 | *X*^2^ = 9.97, *df* = 2 | 0.007 |
|  | 2023 | *X*^2^ =71.84, *df* = 2 | 0.698 |
| ***MRI scan*** | 2003 | *X*^2^ = 20.09, *df* = 2 | < 0.001 |
|  | 2023 | *X*^2^ = 8.7, df = 2 | 0.013 |
| ***ESR*** | 2003 | *X*^2^ = 6.66, *df* = 2 | 0.036 |
|  | 2023 | *X*^2^ = 4.50, *df* = 2 | 0.105 |
| ***Activity*** | 2003 | *X*^2^ = *48.08, df = 2* | < 0.001 |
|  | 2023 | *X*^2^ = *174.91, df = 2* | < 0.001 |
| ***Work*** | 2003 | *X*^2^ = *151.64, df = 2* | < 0.001 |
|  | 2023 | *X*^2^ = *4.62, df = 2* | 0.10 |
| ***Bed-rest*** | 2003 | *X*^2^ = *258.41, df = 2* | < 0.001 |
|  | 2023 | *X*^2^ = *34.13, df = 2* | < 0.001 |
| ***Acupuncture*** | 2003 | *X*^2^ = *2.01, df* = 2 | 0.36 |
|  | 2023 | *X*^2^ = *14.85*, *df* = 2 | < 0.001 |
| ***Massage*** | 2003 | *X*^2^ = *632.78*, *df* = 2 | < 0.001 |
|  | 2023 | *X*^2^ = *158.97*, *df* = 2 | < 0.001 |
| ***Spinal manipulation*** | 2003 | *X*^2^ = *829.94*, *df* = 2 | < 0.001 |
|  | 2023 | *X*^2^ = *422.47*, *df* = 2 | < 0.001 |
| ***Spinal mobilization*** | 2003 | *X*^2^ = *263.48*, *df* = 2 | < 0.001 |
|  | 2023 | *X*^2^ = *136.14*, *df* = 2 | < 0.001 |
| ***General exercise*** | 2003 | *X*^2^ = *69.63*, *df* = 2 | < 0.001 |
|  | 2023 | *X*^2^ = *71.03*, *df* = 2 | < 0.001 |
| ***Specific exercises*** | 2003 | *X*^2^ = *179.01*, *df* = 2 | < 0.001 |
|  | 2023 | *X*^2^ = *61.62*, *df* = 2 | < 0.001 |
| ***Stretching*** | 2003 | *X*^2^ = *448.47*, *df* = 2 | < 0.001 |
|  | 2023 | *X*^2^ = *184.51*, *df* = 2 | < 0.001 |
| ***General advice on back care*** | 2003 | *X*^2^ = *73.59*, *df* = 2 | < 0.001 |
|  | 2023 | *X*^2^ = *3.61*, *df* = 2 | 0.17 |
| ***Address psychosocial issues*** | 2003 | *X*^2^ = *84.64*, *df* = 2 | < 0.001 |
|  | 2023 | *X*^2^ = *15.40*, *df* = 2 | < 0.001 |

*X*^2^ = Chi-square

*df* = degrees of freedom

**Table S4. Less popular investigations selected in 2003 and 2023**

Rate ratios > 1 indicate more frequent selection in 2023 relative to 2003.

| **Investigation** | **Group** | **2003 uptake**  **(%)** | **2023 uptake**  **(%)** | **Rate ratio**  **(95% CI)** | **Adjusted**  ***p*-value** |
| --- | --- | --- | --- | --- | --- |
| ***Sacroiliac X-ray*** | all | 36/1758  (2.05) | 12/1387  (0.87) | 0.422  (0.221, 0.808) | 0.008 |
|  | chiropractors | 13/332  (3.92) | 3/255  (1.18) | 0.3  (0.087, 1.043) | 0.105 |
|  | osteopaths | 14/592  (2.36) | 4/621  (0.64) | 0.272  (0.090, 0.823) | 0.049 |
|  | physiotherapists | 9/834  (1.08) | 5/511  (0.98) | 0.907  (0.306, 2.691) | 1 |
| ***MRI scan*** | all | 18/1758  (1.02) | 40/1387  (2.88) | 2.815  (1.621, 4.887) | < 0.001 |
|  | chiropractors | 1/332  (0.3) | 5/255  (1.96) | 6.51  (0.765, 55.375) | 0.115 |
|  | osteopaths | 15/592  (2.53) | 27/621  (4.35) | 1.716  (0.922, 3.193) | 0.115 |
|  | physiotherapists | 2/834  (0.24) | 8/511  (1.57) | 6.528  (1.392, 30.622) | 0.025 |
| ***CT scan*** | all | 1/1758  (0.06) | 0/1387  (0) | 0  (0, 0) | 1 |
|  | chiropractors | 0/332  (0) | 0/255  (0) | - | 1 |
|  | osteopaths | 1/592  (0.17) | 0/621  (0) | - | 1 |
|  | physiotherapists | 0/834  (0) | 0/511  (0) | - | 1 |
| ***Bone scan*** | all | 3/1758  (0.17) | 1/1387  (0.07) | 0.422  (0.008, 5.261) | 0.635 |
|  | chiropractors | 0/332  (0) | 0/255  (0) | - | 1 |
|  | osteopaths | 3/592  (0.51) | 1/621  (0.16) | 0.318  (0.033, 3.046) | 1 |
|  | physiotherapists | 0/834  (0) | 0/511  (0) | - | 1 |
| ***ESR*** | all | 7/1758  (0.4) | 11/1387  (0.79) | 1.990  (0.774, 5.121) | 0.16 |
|  | chiropractors | 2/332  (0.6) | 4/255  (1.57) | 2.604  (0.481, 14.105) | 0.617 |
|  | osteopaths | 5/592  (0.84) | 6/621  (0.97) | 1.144  (0.351, 3.728) | 1 |
|  | physiotherapists | 0/834  (0) | 1/511  (0.2) | ∞  (∞, ∞) | 0.617 |
| ***Urinalysis*** | all | 12/1758  (0.683) | 9/1387  (0.512) | 0.950  (0.401, 2.248) | 1 |
|  | chiropractors | 8/332  (2.410) | 6/255  (2.353) | 0.976  (0.343, 2.779) | 1 |
|  | osteopaths | 3/592  (0.510) | 2/621  (0.322) | 0.636  (0.1071, 3.790) | 1 |
|  | physiotherapists | 1/834  (0.120) | 1/511  (0.196) | 1.632  (0.102, 26.036) | 1 |
| ***Myelogram*** | all | 6 | 0 | 0 | 0.038 |
|  | chiropractors | 0 | 0 | NA | 1 |
|  | osteopaths | 0 | 0 | NA | 1 |
|  | physiotherapists | 6 | 0 | 0 | 0.266 |
| ***EMG*** | all | 0 | 0 | NA | 1 |
|  | chiropractors | 0 | 0 | NA | 1 |
|  | osteopaths | 0 | 0 | NA | 1 |
|  | physiotherapists | 0 | 0 | NA | 1 |

**Table S5. Less popular interventions selected in 2003 and 2023**

Rate ratios > 1 indicate more frequent selection in 2023 relative to 2003.

| **Intervention** | **Group** | **2003 uptake**  **(%)** | **2023 uptake**  **(%)** | **Rate ratio**  **(95% CI)** | **Adjusted**  ***p*-value** |
| --- | --- | --- | --- | --- | --- |
| ***Neural***  ***mobilization*** | all | 37/1758  (2.10) | 26/1387  (1.87) | 0.89  (0.542, 1.462) | 0.702 |
|  | chiropractors | 7/332  (2.11) | 5/255  (1.96) | 0.93  (0.299, 2.896) | 1 |
|  | osteopaths | 18/592  (3.04) | 15/621  (2.42) | 0.794  (0.404, 1.562) | 1 |
|  | physio | 12/834  (1.44) | 6/511  (1.17) | 0.816  (0.308, 2.161) | 1 |
| ***Traction*** | all | 59/1758  (3.36) | 45/1387  (3.24) | 0.966  (0.66, 1.415) | 0.92 |
|  | chiropractors | 8/332  (2.41) | 10/255  (3.92) | 1.627  (0.652, 4.064) | 0.508 |
|  | osteopaths | 48/592  (8.11) | 32/621  (5.15) | 0.636  (0.412, 0.98) | 0.146 |
|  | physiotherapists | 3/834  (0.36) | 3/511  (0.59) | 1.632  (0.331, 8.056) | 0.679 |
| ***Corset*** | all | 50/1758  (2.84) | 34/1387  (2.45) | 0.861  (0.56, 1.324) | 0.507 |
|  | chiropractors | 7/332  (2.11) | 10/255  (3.92) | 1.86  (0.718, 4.819) | 0.330 |
|  | osteopaths | 35/592  (5.91) | 19/621  (3.06) | 0.518  (0.299, 0.894) | 0.053 |
|  | physiotherapists | 8/834  (0.96) | 5/511  (0.98) | 1.02  (0.336, 3.101) | 1 |
| ***TENS*** | all | 49/1758  (2.79) | 23/1387  (1.66) | 0.595  (0.364, 0.971) | 0.041 |
|  | chiropractors | 1/332  (0.3) | 2/255  (0.78) | 2.604  (0.237, 28.558) | 0.582 |
|  | osteopaths | 11/592  (1.86) | 5/621  (0.81) | 0.433  (0.151, 1.24) | 0.377 |
|  | physiotherapists | 37/834  (4.44) | 16/511  (3.13) | 0.706  (0.397, 1.256) | 0.377 |

**Table S6. Inter-professional comparisons of investigations in 2003 and 2023**

Rate ratios > 1 indicate more frequent selection by Group 2 relative to Group 1.

| **Investigation** | **Year** | **Group 1** | **Group 2** | **Effect size** | **Adjusted**  ***p*-value** |
| --- | --- | --- | --- | --- | --- |
| ***No investigations*** | 2003 | physio | chiro | RR = 0.867  (0.816, 0.922) | < 0.001 |
|  |  | osteo | chiro | RR = 0.96  (0.896, 1.028) | 0.228 |
|  |  | osteo | physio | RR = 1.107  (1.059, 1.157) | < 0.001 |
|  | 2023 | physio | chiro | 0.946  (0.895, 1) | 0.067 |
|  |  | osteo | chiro | 0.992  (0.937, 1.051) | 0.826 |
|  |  | osteo | physio | 1.049  (1.007, 1.092) | 0.067 |
| ***ESR*** | 2003 | physio | chiro | RR = Inf  (Inf, Inf) | 0.121 |
|  |  | osteo | chiro | RR = 0.713  (0.139, 3.656) | 1 |
|  |  | osteo | physio | RR = 0  (0, 0) | 0.037 |
|  | 2023 | physio | chiro | RR = 8.016  (0.901, 71.346) | 0.134 |
|  |  | osteo | chiro | RR = 1.624  (0.462, 5.705) | 0.488 |
|  |  | osteo | physio | RR = 0.203  (0.024, 1.677) | 0.204 |
| ***Urinalysis*** | 2003 | physio | chiro | RR = 20.096  (2.523, 160.052) | < 0.001 |
|  |  | osteo | chiro | RR = 4.755  (1.27, 17.801) | 0.032 |
|  |  | osteo | physio | RR = 0.237  (0.025, 2.269) | 0.313 |
|  | 2023 | physio | chiro | 12.024  (1.455, 99.338) | 0.014 |
|  |  | osteo | chiro | 7.306  (1.484, 35.957) | 0.014 |
|  |  | osteo | physio | 0.608  (0.055, 6.682) | 1 |
| ***Lumbosacral X-ray*** | 2003 | physio | chiro | RR = 3.205  (2.005, 5.124) | < 0.001 |
|  |  | osteo | chiro | RR = 2.062  (1.31, 3.245) | 0.004 |
|  |  | osteo | physio | RR = 0.643  (0.394, 1.051) | 0.085 |
|  | 2023 | physio | chiro | 2.405  (1.053, 5.491) | 0.059 |
|  |  | osteo | chiro | 3.653  (1.511, 8.83) | 0.013 |
|  |  | osteo | physio | 1.519  (0.604, 3.821) | 0.475 |
| ***Sacroiliac X-ray*** | 2003 | physio | chiro | RR = 3.629  (1.566, 8.408) | 0.009 |
|  |  | osteo | chiro | RR = 1.656  (0.788, 3.48) | 0.221 |
|  |  | osteo | physio | RR = 0.456  (0.199, 1.047) | 0.128 |
|  | 2023 | physio | chiro | RR = 1.202  (0.29, 4.991) | 1 |
|  |  | osteo | chiro | RR = 1.826  (0.412, 8.103) | 1 |
|  |  | osteo | physio | RR = 1.519  (0.41, 5.628) | 1 |
| ***MRI scan*** | 2003 | physio | chiro | RR = 1.256  (0.114, 13.805) | 1 |
|  |  | osteo | chiro | RR = 0.119  (0.016, 0.896) | 0.022 |
|  |  | osteo | physio | RR = 0.095  (0.022, 0.412) | < 0.001 |
|  | 2023 | physio | chiro | 1.252  (0.414, 3.79) | 0.768 |
|  |  | osteo | chiro | 0.451  (0.176, 1.158) | 0.168 |
|  |  | osteo | physio | 0.36  (0.165, 0.786) | 0.026 |

RR = risk ratio (95% CI)

**Table S7. Inter-professional comparisons of interventions in 2003 and 2023**

Rate ratios > 1 indicate more frequent selection by Group 2 relative to Group 1.

| **Intervention** | **Year** | **Group 1** | **Group 2** | **Effect size** | **Adjusted**  ***p*-value** |
| --- | --- | --- | --- | --- | --- |
| ***Activity*** | 2003 | physio | chiro | *Z* = -6.514 | < 0.001 |
|  |  | osteo | chiro | *Z* = 3.363 | < 0.001 |
|  |  | osteo | physio | *Z* = -12.157 | < 0.001 |
|  | 2023 | physio | chiro | *Z* = -4.776 | < 0.001 |
|  |  | osteo | chiro | Z = 0.117 | 0.453 |
|  |  | osteo | physio | *Z* = -6.277 | < 0.001 |
| ***Work*** | 2003 | physio | chiro | *Z* = -5.723 | < 0.001 |
|  |  | osteo | chiro | *Z* = 3.476 | < 0.001 |
|  |  | osteo | physio | *Z* = -11.346 | < 0.001 |
|  | 2023 | physio | chiro | *Z* = -1.979 | 0.072 |
|  |  | osteo | chiro | *Z* = -0.474 | 0.318 |
|  |  | osteo | physio | *Z* = -1.950 | 0.038 |
| ***Bed-rest*** | 2003 | physio | chiro | *Z* = -4.967 | < 0.001 |
|  |  | osteo | chiro | *Z* = 7.077 | < 0.001 |
|  |  | osteo | physio | *Z* = -15.027 | < 0.001 |
|  | 2023 | physio | chiro | *Z* = -3.144 | 0.001 |
|  |  | osteo | chiro | *Z* = 1.155 | 0.16 |
|  |  | osteo | physio | *Z* = -5.528 | < 0.001 |
| ***Acupuncture*** | 2003 | physio | chiro | RR = 0.718  (0.432, 1.193) | 0.375 |
|  |  | osteo | chiro | RR = 0.698  (0.411, 1.183) | 0.375 |
|  |  | osteo | physio | RR = 0.972  (0.675, 1.401) | 0.92 |
|  | 2023 | physio | chiro | RR = 1.555  (1.119, 2.162) | 0.016 |
|  |  | osteo | chiro | RR = 0.938  0.706, 1.247) | 0.717 |
|  |  | osteo | physio | RR = 0.603  0.461, 0.789) | 0.001 |
| ***Massage*** | 2003 | physio | chiro | RR = 3.102  (2.676, 3.596) | < 0.001 |
|  |  | osteo | chiro | RR = 0.779  (0.72, 0.844) | < 0.001 |
|  |  | osteo | physio | RR = 0.251  (0.22, 0.287) | < 0.001 |
|  | 2023 | physio | chiro | RR = 1.49  (1.317, 1.686) | < 0.001 |
|  |  | osteo | chiro | RR = 0.847  (0.774, 0.926) | < 0.001 |
|  |  | osteo | physio | RR = 0.568  (0.514, 0.628) | < 0.001 |
| ***Spinal manipulation*** | 2003 | physio | chiro | RR = 13.368  (10.353, 17.26) | < 0.001 |
|  |  | osteo | chiro | RR = 1.476  (1.371, 1.59 | < 0.001 |
|  |  | osteo | physio | RR = 0.11  (0.085, 0.143) | < 0.001 |
|  | 2023 | physio | chiro | RR = 22.633  (14.108, 36.308) | < 0.001 |
|  |  | osteo | chiro | RR = 1.973  (1.746, 2.23) | < 0.001 |
|  |  | osteo | physio | RR = 0.087  (0.054, 0.141) | < 0.001 |
| ***Spinal mobilization*** | 2003 | physio | chiro | RR = 0.33  (0.257, 0.424) | < 0.001 |
|  |  | osteo | chiro | RR = 0.23  (0.179, 0.294) | < 0.001 |
|  |  | osteo | physio | RR = 0.697  (0.64, 0.758) | < 0.001 |
|  | 2023 | physio | chiro | RR = 1.324  (1.102, 1.592) | 0.013 |
|  |  | osteo | chiro | RR = 0.655  (0.565, 0.76) | < 0.001 |
|  |  | osteo | physio | RR = 0.495  (0.433, 0.566) | < 0.001 |
| ***Stretching*** | 2003 | physio | chiro | RR = 2.335  (1.912, 2.852) | < 0.001 |
|  |  | osteo | chiro | RR = 0.546  (0.474, 0.629) | < 0.001 |
|  |  | osteo | physio | RR = 0.234  (0.2, 0.274) | < 0.001 |
|  | 2023 | physio | chiro | RR = 1.7  (1.389, 2.081) | < 0.001 |
|  |  | osteo | chiro | RR = 0.664  (0.571, 0.771) | < 0.001 |
|  |  | osteo | physio | RR = 0.39  (0.333, 0.457) | < 0.001 |
| ***Traction*** | 2003 | physio | chiro | RR = 6.699  (1.788, 25.096) | 0.009 |
|  |  | osteo | chiro | RR = 0.297  (0.142, 0.621) | < 0.001 |
|  |  | osteo | physio | RR = 0.044  (0.014, 0.142) | < 0.001 |
|  | 2023 | physio | chiro | RR = 6.68  (1.855, 24.059) | 0.004 |
|  |  | osteo | chiro | RR = 0.761  (0.38, 1.525) | 1 |
|  |  | osteo | physio | RR = 0.114  (0.035, 0.37) | < 0.001 |
| ***General advice*** | 2003 | physio | chiro | RR = 0.822  (0.769, 0.878) | < 0.001 |
|  |  | osteo | chiro | RR = 0.985  (0.912, 1.064) | 1 |
|  |  | osteo | physio | RR = 1.198  (1.14, 1.26) | < 0.001 |
|  | 2023 | physio | chiro | RR = 1.108  (0.998, 1.23) | 0.194 |
|  |  | osteo | chiro | RR = 1.055  (0.956, 1.164) | 0.342 |
|  |  | osteo | physio | RR = 0.952  (0.873, 1.039) | 0.342 |
| ***General exercise*** | 2003 | physio | chiro | RR = 0.609  (0.512, 0.724) | < 0.001 |
|  |  | osteo | chiro | RR = 0.982  (0.805, 1.199) | 1 |
|  |  | osteo | physio | RR = 1.613  (1.408, 1.848) | < 0.001 |
|  | 2023 | physio | chiro | RR = 0.529  (0.431, 0.65) | < 0.001 |
|  |  | osteo | chiro | RR = 0.862  (0.692, 1.073) | 0.537 |
|  |  | osteo | physio | RR = 1.628  (1.424, 1.861) | < 0.001 |
| ***Specific exercises*** | 2003 | physio | chiro | RR = 0.439  (0.371, 0.519) | < 0.001 |
|  |  | osteo | chiro | RR = 0.686  (0.571, 0.826) | < 0.001 |
|  |  | osteo | physio | RR = 1.564  (1.416, 1.729) | < 0.001 |
|  | 2023 | physio | chiro | RR = 0.471  (0.377, 0.588) | < 0.001 |
|  |  | osteo | chiro | RR = 0.635  (0.506, 0.798) | < 0.001 |
|  |  | osteo | physio | RR = 1.35  (1.193, 1.527) | < 0.001 |
| ***Address psychosocial issues*** | 2003 | physio | chiro | RR = 0.592  (0.483, 0.726) | < 0.001 |
|  |  | osteo | chiro | RR = 1.239  (0.967, 1.587) | 0.289 |
|  |  | osteo | physio | RR = 2.093  (1.748, 2.507) | < 0.001 |
|  | 2023 | physio | chiro | RR = 0.608  (0.462, 0.801) | < 0.001 |
|  |  | osteo | chiro | RR = 0.771  (0.584, 1.02) | 0.205 |
|  |  | osteo | physio | RR = 1.268  (1.057, 1.521) | 0.037 |
| ***Corset*** | 2003 | physio | chiro | RR = 2.198  (0.803, 6.013) | 0.442 |
|  |  | osteo | chiro | RR = 0.357  (0.16, 0.794) | 0.023 |
|  |  | osteo | physio | RR = 0.162  (0.076, 0.347) | < 0.001 |
|  | 2023 | physio | chiro | RR = 4.008  (1.384, 11.602) | 0.03 |
|  |  | osteo | chiro | RR = 1.282  (0.604, 2.718) | 1 |
|  |  | osteo | physio | RR = 0.32  (0.12, 0.851) | 0.062 |
| ***Interferential*** | 2003 | physio | chiro | RR = 0.748  (0.5, 1.119) | 0.253 |
|  |  | osteo | chiro | RR = 1.248  (0.785, 1.985) | 0.36 |
|  |  | osteo | physio | RR = 1.668  (1.17, 2.378) | 0.013 |
|  | 2023 | physio | chiro | RR = 0.891  (0.277, 2.864) | 1 |
|  |  | osteo | chiro | RR = 1.392  (0.411, 4.713) | 1 |
|  |  | osteo | physio | RR = 1.562  (0.586, 4.166) | 1 |
| ***Ultrasound*** | 2003 | physio | chiro | RR = 0.98  (0.72, 1.334) | 0.927 |
|  |  | osteo | chiro | RR = 1.427  (1, 2.034) | 0.082 |
|  |  | osteo | physio | RR = 1.455  (1.089, 1.945) | 0.031 |
|  | 2023 | physio | chiro | RR = 0.668  (0.319, 1.399) | 0.599 |
|  |  | osteo | chiro | RR = 0.843  (0.401, 1.774) | 0.709 |
|  |  | osteo | physio | RR = 1.262  (0.746, 2.135) | 0.599 |
| ***TENS*** | 2003 | physio | chiro | RR = 0.068  (0.009, 0.493) | < 0.001 |
|  |  | osteo | chiro | RR = 0.162  (0.021, 1.25) | 0.065 |
|  |  | osteo | physio | RR = 2.388  (1.228, 4.642) | 0.011 |
|  | 2023 | physio | chiro | RR = 0.25  (0.058, 1.081) | 0.067 |
|  |  | osteo | chiro | RR = 0.974  (0.19, 4.988) | 1 |
|  |  | osteo | physio | RR = 3.889  (1.434, 10.543) | 0.019 |

RR = risk ratio (95% CI)

*Z* = Z-score

**Table S8. Characteristics of clinicians included in the sensitivity analysis**

Clinicians matched to smallest group per year (chiropractors in both 2003 and 2023) by gender, age, and years qualified

| **Characteristic** | **Group** | **2003** | **2023** | ***P*-value** |
| --- | --- | --- | --- | --- |
| Female gender, n (%) | all | 451/984 (45.8) | 401/759 (52.8) | 0.004 |
|  | chiropractors | 124/328 (37.8) | 134/253 (53.0) | < 0.001 |
|  | osteopaths | 124/328 (37.8) | 134/253 (53.0) | <0.001 |
|  | physiotherapists | 203/328 (61.9) | 133/253 (52.6) | 0.030 |
| Age in years, mean (95% CI) | all | 39.0 (38.5, 39.6) | 45.3 (44.4, 46.2) | < 0.001 |
|  | chiropractors | 38.7 (37.6, 39.7) | 45.6 (43.9, 47.3) | < 0.001 |
|  | osteopaths | 39.1 (38.1, 40.1) | 45.4 (43.7, 47.0) | < 0.001 |
|  | physiotherapists | 49.3 (38.4, 40.3) | 45.0 (43.6, 46.4) | < 0.001 |
| Years qualified, mean (95% CI) | all | 12.1 (11.6, 12.6) | 16.8 (16.0, 17.6) | < 0.001 |
|  | chiropractors | 10.7 (9.87, 11.6) | 16.3 (14.9, 17.7) | < 0.001 |
|  | osteopaths | 11.1 (10.3, 11.9) | 16.3 (14.9, 17.7) | < 0.001 |
|  | physiotherapists | 14.4 (13.6, 15.2) | 17.8 (16.5, 19.1) | < 0.001 |
| Lived LBP experience ever, n (%) | all | 780/984 (79.3) | 622/759 (81.9) | 0.181 |
|  | chiropractors | 273/328 (83.2) | 213/253 (84.2) | 0.844 |
|  | osteopaths | 279/328 (85.1) | 210/253 (83.0) | 0.576 |
|  | physiotherapists | 228/328 (69.5) | 199/253 (78.7) | 0.017 |
| Provides care for NHS patients, n (%) | all | 297/983 (30.2) | 122/759 (16.1) | < 0.001 |
|  | chiropractors | 31/327 (9.5) | 3/253 (1.2) | < 0.001 |
|  | osteopaths | 37/328 (11.3) | 11/253 (4.3) | 0.004 |
|  | physiotherapists | 229/328 (69.8) | 108/253 (42.7) | < 0.001 |
